# Association of Ultraprocessed Foods Intake with Untargeted Metabolomics Profiles in Adolescents and Young Adults in the DONALD Cohort Study

**DOI:** 10.1016/j.tjnut.2024.09.023

**Published:** 2024-09-25

**Authors:** Samuel Muli, Annika Blumenthal, Christina-Alexandra Conzen, Maike Elena Benz, Ute Alexy, Matthias Schmid, Pekka Keski-Rahkonen, Anna Floegel, Ute Nöthlings

**Affiliations:** 1 Unit of Nutritional Epidemiology, Department of Nutrition and Food Sciences, University of Bonn, Bonn, Germany; 2 Institute for Medical Biometry, Informatics and Epidemiology (IMBIE), University Hospital Bonn, Bonn, Germany; 3 International Agency for Research on Cancer (IARC), Lyon, France; 4 Section of Dietetics, Faculty of Agriculture and Food Sciences, Hochschule Neubrandenburg, Neubrandenburg, Germany

**Keywords:** ultraprocessed foods, dietary biomarkers, metabolomics, metabolites, metabolite patterns

## Abstract

**Background:**

High consumption of ultraprocessed foods (UPFs) continues to draw significant public health interest because of the associated negative health outcomes. Metabolomics can contribute to the understanding of the biological mechanisms through which UPFs may influence health.

**Objectives:**

To investigate urine and plasma metabolomic biomarkers of UPF intake in adolescents and young adults.

**Methods:**

We used data from the Dortmund Nutritional and Anthropometric Longitudinally Designed study to investigate cross-sectional associations of UPF intake with concentrations of urine metabolites in adolescents using 3d weighed dietary records (3d-WDR) and 24-h urine samples (*n* = 339), and associations of repeatedly assessed UPF intake with concentrations of circulating plasma metabolites in young adults with 3–6 3d-WDRs within 5 y preceding blood measurement (*n* = 195). Urine and plasma samples were analyzed using mass spectrometry-based metabolomics. Biosample-specific metabolite patterns (MPs) were determined using robust sparse principal components analysis. Multivariable linear regression models were applied to assess the associations of UPF consumption (as a percentage of total food intake in g/d) with concentrations of individual metabolites and MP scores.

**Results:**

The median proportion of UPF intake was 22.0% [interquartile range (IQR): 12.3, 32.9] in adolescents and 23.2% (IQR: 16.0, 31.6) in young adults. We identified 42 and 6 UPF intake-associated metabolites in urine and plasma samples, respectively. One urinary MP, “xenobiotics and amino acids” [*β* = 0.042, 95% confidence interval (CI): 0.014, 0.070] and 1 plasma MP, “lipids, xenobiotics, and amino acids” (*β* = 0.074, 95% CI: 0.031, 0.117) showed positive association with UPF intake. Both patterns shared 29 metabolites, mostly of xenobiotic metabolism.

**Conclusions:**

We identified urine and plasma metabolites associated with UPF intake in adolescents and young adults, which may represent some of the biological mechanisms through which UPFs may influence metabolism and health.

## Introduction

Industrial food processing, which combines ingredients and additives, is important for improving food safety, nutritional access, and in reducing food waste across the supply chain [1]. Ultraprocessed foods (UPFs) as defined by the NOVA classification system [2], includes a broad range of industrially processed food products such as soft drinks, flavored yogurts, packaged snacks, confectionery, pasta and pizza dishes, processed meat products, instant noodles, soups and sauces, among other ready-to-heat or -eat food products [[2], [3], [4]]. Broadly, most UPFs contain a mix of food-derived or reconstituted ingredients and other industrially isolated components such as lactose, casein, gluten, whey, hydrogenated oils, variety of sugars (e.g., high fructose corn syrup), cosmetic additives such as colorants, dyes, flavors enhancers, bulking agents, emulsifiers among others, mostly of exclusive industrial use [2,3,5]. These formulations provide convenience, affordability, and enhance shelf-life and sensory properties [2].

The consumption of UPFs has been rising, contributing to more than half of the total daily energy intake in some countries, for example, the United States [6]. In 22 European countries, UPF intake varies markedly, ranging from 14% to 44%, as reported in a recent study [7]. An increasing number of epidemiologic studies suggest that high consumption of UPFs is associated with increased health risks—including obesity [8], cardiometabolic diseases [4,9], chronic kidney disease [10], cancer [9,11], irritable bowel syndrome [12], and depression [13]. Some of these health risks have been shown to be particularly significant for higher intake of animal-based products and sweetened beverages [9].

Several mechanisms have been proposed to explain ways through which UPFs may influence health. These include poor nutrient profiles: excessive added sugars, salts, unhealthy fats, and high energy density but low in protein and dietary fiber [2,14]. Furthermore, the physical and chemical properties associated with industrial processing, ingredients, and their by-products may also contribute to these increased health risks [4]. Nevertheless, the debate, widespread disagreement, and uncertainty on the links between UPFs and increased health risks still exists [[15], [16], [17], [18], [19]].

Untargeted metabolomics is a promising approach for investigating the relationship between the consumption of UPFs and health status, as dietary intakes elicit metabolic changes that can be related to health indicators. Urine and blood matrices may reflect different aspects of dietary intake and metabolic changes, with urine more reliably reflecting short-term changes in concentrations of diet-responsive metabolites, and blood a more stable overview of an individual’s metabolic state [20]. These insights could improve our understanding of pathways through which UPF may affect health, encouraging more nuanced discourses on these biological mechanisms. Indeed, some metabolomics-based studies have investigated the links between individual foods within the UPFs such as sweetened beverages [21], processed red meats [22,23], and metabolic health. Such studies are valuable, particularly in identifying biomarkers associated with the intake of specific foods. However, considering UPFs as an aggregate dietary pattern may more accurately reflect actual dietary habits, as foods and nutrients are typically consumed in combinations [[24], [25], [26]].

So far, the associations of various dietary patterns and metabolomics profiles have been extensively described [27], but studies on UPF-metabolome associations are markedly fewer. This gap is evident across all age groups, with only a handful of studies conducted in adults [10,28,29] and in younger populations [30,31]. Therefore, our study investigates the association between UPF intake and untargeted urine and plasma metabolomics profiles in adolescents and young adults from a well-characterized German cohort.

## Methods

### Study design and population

The Dortmund Nutritional and Anthropometric Longitudinally Designed (DONALD) study is an open dynamic cohort on children and young individuals residing in Dortmund, Germany. Since 1985, 30–40 healthy infants are recruited annually during their first year and are regularly assessed and followed up until adulthood. Regular assessments include dietary intake, anthropometric measurements, urine sample collection (from the ages 3–4 y), blood samples (from 18 y), medical, lifestyle, and other sociodemographic data [32].

The DONALD study was approved by the Ethics Committee of the University of Bonn (ethics numbers: 098/06 and 185/20). All procedures and assessments followed ethical standards of the Declaration of Helsinki, with written informed consent from parents and from adolescents from the age of 16 y.

### Study sample

The current analyses included 2 analytic samples termed adolescent urine *n* = 339, and young adult plasma *n* = 195, previously described in [33]. In brief, the adolescent urine sample included individuals who provided a 3-d weighed dietary record (3d-WDR) and a single 24-h urine sample (age at urine sample, 14.9–18.4 y). The young adult plasma sample included individuals who completed 3 or more 3d-WDRs within the 5-y period preceding a single blood measurement (age at blood sample, 18.0–21.9 y). The 2 analytic samples had an overlap of 139 participants. A study flowchart is provided in Supplemental Figure 1.

### Dietary assessment

In the 3d-WDRs, the study participants, or with parental assistance, weighed all foods and beverages consumed and leftovers to the nearest 1 g using electronic food scales. Semiquantitative estimates (e.g., portion sizes, cups, or spoons) were acceptable if weighing the food was not possible such as for meals consumed away from home. Information on recorded food products, including brands, ingredients, declared nutrients, and methods of preparation were also collected. Each product recorded for the first time receives its own entry in an internally maintained and regularly updated food composition in-house database (LEBTAB) [34]. The nutritional profiles of staple foods were derived from German food composition tables, whereas the caloric and nutrient content of packaged food products (e.g., processed items, convenience meals, and snacks) were determined through recipe simulation from their ingredients and nutrients labels.

### Food and beverage groupings

All food and beverage items recorded by the participants were assigned into 1 of the NOVA categories according to the purpose, nature, and extent of their processing [2]: NOVA-1 [unprocessed or minimally processed foods (UMFs)], NOVA-2 (processed culinary ingredients), NOVA-3 (processed foods), and NOVA-4 (UPF). Food items with unclear NOVA classification were documented and classified on the basis of internal consensus and the Federal Ministry of Food and Agriculture guideline for spices and other seasonings [35]. A summary of food and beverage groups according to the NOVA system is provided in Supplemental Table 1.

This study primarily focused on the UPFs. Daily intakes were calculated as individual means from the 3d-WDR. We defined the long-term UPF intake variable as the mean intake across all 3d-WDRs within the 5-y period preceding blood draw. In our main analysis, we calculated the proportion of UPF intake as a percentage of total weight of food and beverages consumed in grams. This weight-based ratio instead of the energy-based ratio acknowledges food and beverages that provide low or zero calories [11,36,37] as well as nonnutritive ingredients and additives that may be used in food processing [37].

### Other covariate assessment

Experienced nurses conducted anthropometric measurements following standardized procedures. BMI (kg/m^2^) was calculated from these measurements. Leisure time physical activity was assessed using a questionnaire adapted from the validated Adolescent Physical Activity Recall Questionnaire [38]. Participants estimated the time spent on a range of organized and unorganized sports over the past 12 mo, and the reported activities quantified in metabolic equivalent of task-hours per week. Self-reported alcohol use and smoking status were assessed using a questionnaire, and participants categorized as current, former, and never for each of these lifestyle factors. The covariate data represent measurements closest to or on the date of biosample collection.

### Urine and blood samples

Following a standardized protocol, participants collected their 24-h urine samples on the third day of the 3-d dietary assessment. These were then stored in sterile, preservative-free plastic containers at temperatures below −12°C. Upon transfer to the DONALD study center, the urine samples were stored at −22°C until processed. Fasting blood sample were drawn, centrifuged at 4°C for 15 min (3100 U/min, 2000 × *g*), aliquoted and stored at −80°C. EDTA plasma was used in this analysis. Detailed procedures for urine and blood samples are provided [32].

### Metabolite profiling

Metabolon Inc. conducted untargeted metabolomics analysis on the urine and plasma samples using ultrahigh performance liquid chromatography-tandem mass spectroscopy. Metabolon followed their standardized protocol for sample handling, raw data extraction, and peak identification and analysis as outlined in their procedures [39]. For plasma samples, Metabolon applied both metabolomics and lipidomics approaches. Overall, 1407 metabolites were annotated in urine and 1042 features in plasma samples. We provide a detailed description of the metabolomics procedures for both urine and blood samples in the Supplemental Methods.

### Statistical analyses

#### Descriptive statistics

Characteristics of study participants were expressed as medians (25th and 75th percentile) for continuous variables and frequencies (percentages) for categorical variables.

#### Processing of metabolite data

We performed mechanism aware imputation of missing values in 2 steps. First, we applied a novel method that combines particle swarm optimization (to search for metabolite concentration thresholds and the proportion of low concentration deletions) and extreme gradient boosting as classifier method for mechanism underlying each missing value, following procedures provided by Yuan et al. [40]. These were implemented in Python using NumPy, Pandas, scikit-learn, and XGBoost libraries. Our data predominantly showed missing not at random values in urine (86.1%) and in plasma (83.2%) samples. Therefore, metabolites with >20% missing data were excluded according to the “80% rule” [41], and the rest were imputed by quantile regression imputation of left-censored data using MetImp 1.2 [42]. Batch normalization were conducted using *ber* bagging method implemented in the dbnorm R package [43]. Subsequently, the data were natural log-transformed, mean centered, and scaled to unit variance.

#### Deriving metabolite patterns

We used the robust sparse principal component analysis (Robust SPCA) to compute the metabolite patterns (MPs) because of better interpretability of its components through sparse vectors, as the loadings are determined from a subset of the original variables, and its robustness to outlying observations [44]. We implemented these steps using the sparsepca R package. The optimal PCA components retained were determined by scree plots using the PCAtools R package.

#### Associations of UPF with metabolites and MPs

Using multivariable linear regression, we regressed *1*) each of the single metabolites and *2*) each of the MPs on the UPF intake, adjusting for age, sex, energy intake, BMI, physical activity, smoking, and alcohol status. The plasma models were additionally adjusted for the time difference between dietary assessments and blood draw (time difference = age at blood draw − mean age of dietary assessments) and the number of dietary assessments per participant. We applied the Benjamini–Hochberg procedure to control the false discovery rate at 5% within each set of regression analyses. For the main results, we assessed model assumptions (i.e., normality of residuals, linearity, and homogeneity of variance) using the performance R package.

#### Missing covariates

Considering the DONALD’s longitudinal design, we first applied backward filling for “never” alcohol intake (or smokers) to fill missing data for earlier time points. We then imputed the rest of missing data for physical activity, alcohol use, and smoking status (Table 1) using the K-nearest neighbor algorithm, with 10 nearest neighbors on the basis of other nonmissing covariate data. These were implemented using the VIM R package.

**TABLE 1:** Basic characteristics of the study participants.

	*n*	Adolescent urine (*N* = 339)	*n*	Young adult plasma (*N* = 195)
Sex: female	339	166 (49.0)	195	108 (55.4)
Age at biosample collection (y)	339	18.0 (17.0, 18.1)	195	18.1 (18.1, 18.2)
BMI (kg/m^2^)	339	21.9 (19.9, 24.0)	195	22.2 (20.1, 24.5)
UPF, % total food intake (g/d)	339	22.0 (12.3, 32.9)	195	23.2 (16.0, 31.6)
UPF, % TEI	339	42.0 (32.2, 52.0)	195	45.0 (36.8, 50.8)
Energy intake (TEI, Kcal/d)	339	2126.9 (1748.5, 2582.1)	195	1978.1 (1697.0, 2390.1)
3d-WDR assessments	339	1.0	195	4.0 (4.0, 5.0)
Physical activity (MET-h /w)	215	34.0 (14.1, 54.8)	184	30.1 (12.1, 52.9)
Smoking status	211		142	
Never		155 (73.5)		98 (69.0)
Former		23 (10.9)		21 (14.8)
Current		33 (15.6)		23 (16.2)
Alcohol status	179		153	
Never		24 (13.4)		20 (13.1)
Former		27 (15.1)		31 (20.3)
Current		128 (71.5)		102 (66.7)

Abbreviations: 3d-WDR, 3-d weighed dietary records, MET-h /w, metabolic equivalent of task-hours per week; TEI, total energy intake; UPF, ultraprocessed food.

Data are presented as *n* (%) and median (25th and 75th percentile) for categorical and continuous variables, respectively. Differences in *n* are because of missing data values.

The young adult label reflects age at blood sample collection (min-max. 18.0–21.9 y) but the repeated dietary assessments were conducted over the 5 y preceding the blood draw.

#### Additional analyses

Considering differences in UPF variable definition in literature, such as absolute intakes, weight-based ratio, and energy-based ratio [[9], [10], [11],[28], [29], [30], [31],^36^,^37^], we conducted secondary analysis to compare our main results (weight-based ratio) with absolute (g/d) and energy-based ratio (energy from UPFs as a percentage of total energy intake). We also computed correlation (Pearson) between UPF and UMF intakes to investigate the hypothesis that a higher UPF consumption is related to reduced UMF intake [2,4]. Lastly, we performed sensitivity analyses on urine samples, *n* = 260, and plasma, *n* = 137, after excluding potentially implausible 3d-WDR reporting on the basis of sex- and age-specific thresholds for underreporting [45].

All statistical analyses were conducted using Python (v3.8) and R (v4.1.3).

## Results

### Descriptive characteristics

**FIGURE 1:**
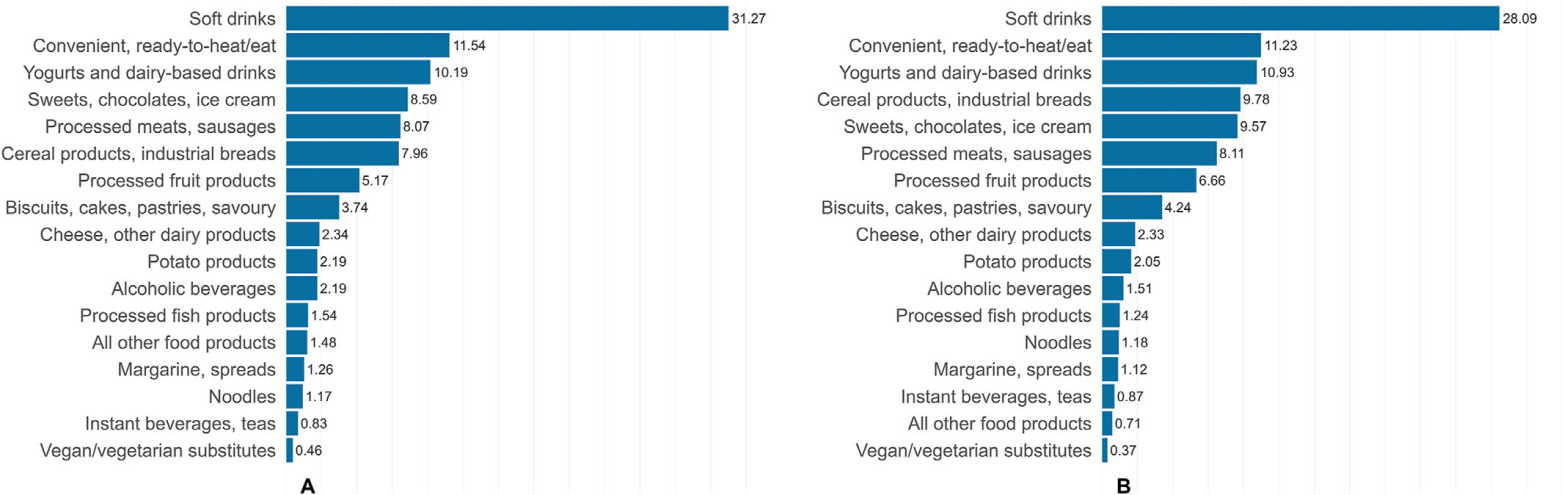
Mean contribution of various food groups to the total UPF consumption, as a percentage of the total weight (g/d) in (A), adolescent urine and (B), young adults’ plasma analytic samples. UPF, ultraprocessed food.

The adolescent urine samples (49.0% female) and young adult plasma samples (55.4% female) had median ages of 18.0 and 18.1 y, with median BMIs of 21.9 and 22.2, respectively. The median proportion of UPF intake to total food intake by weight was 22.0% (IQR: 12.3%, 32.9%) in adolescents and 23.2% (IQR: 16.0%, 31.6%) in young adults (Table 1). The foods with highest mean contribution to total UPF intake in both analytic samples were sweetened beverages (nondairy soft drinks) and convenient, ready-to-heat or -eat food products, contributing 31.3% and 11.5% (adolescents) and 28.1% and 11.2% (young adults), respectively (Figure 1). Regarding energy intake, however, sweets, chocolates, and ice cream; cereals and industrial breads; and processed meats and sausages had the highest energy contribution to the total calorie intake from the UPFs in both adolescents and young adults (Supplemental Figure 2).

### Associations between UPF intake and urine metabolites

In adolescent urine samples, of the 42 urine metabolites identified in our fully adjusted model, 21 metabolites were positively associated with UPF intake (Table 2). In accordance with the Dragsted et al. [46,47] framework for evaluating food biomarker plausibility, which suggests that biomarkers of intake should demonstrate dose–response relationship (i.e., increase in human sample with higher food intake), in this section, we highlight the positive associations of UPF intake and urine metabolites and provide all associations observed in Supplemental Table 2.

**TABLE 2:** Regression estimates of the associations between UPF intake and urine metabolites in adolescents (*n* = 339).

Metabolite	Model 1, *β* (95% CI)	Model 2, *β* (95% CI)	Model 3, *β* (95% CI)
X-17679	0.026 (0.021, 0.032)^1^	0.026 (0.020, 0.032)^1^	0.027 (0.021, 0.033)^1^
X-19497	0.013 (0.007, 0.019)^1^	0.014 (0.008, 0.020)^1^	0.016 (0.010, 0.022)^1^
Glucuronide of C10H18O2 (1)^2^	0.014 (0.007, 0.020)^1^	0.014 (0.007, 0.020)^1^	0.015 (0.008, 0.021)^1^
Glucuronide of C10H14O2 (2)^2^	0.013 (0.007, 0.019)^1^	0.013 (0.007, 0.020)^1^	0.015 (0.008, 0.021)^1^
X-11478	0.012 (0.006, 0.018)^1^	0.013 (0.006, 0.019)^1^	0.014 (0.007, 0.020)^1^
Glucuronide of C10H18O2 (7)^2^	0.012 (0.006, 0.018)^1^	0.012 (0.006, 0.019)^1^	0.014 (0.007, 0.021)^1^
Indoxyl glucuronide	0.005 (–0.001, 0.011)	0.010 (0.004, 0.016)^1^	0.013 (0.007, 0.019)^1^
Glucuronide of C10H18O2 (8)^2^	0.013 (0.007, 0.019)^1^	0.012 (0.006, 0.019)^1^	0.014 (0.007, 0.020)^1^
N,N-dimethylalanine	0.010 (0.004, 0.016)^1^	0.012 (0.006, 0.018)^1^	0.013 (0.007, 0.019)^1^
3-Hydroxy-N6,N6,N6-trimethyl-L-lysine^2^	0.012 (0.006, 0.018)^1^	0.012 (0.006, 0.018)^1^	0.012 (0.006, 0.018)^1^
1-methylhistamine	0.011 (0.005, 0.016)^1^	0.011 (0.005, 0.017)^1^	0.012 (0.006, 0.018)^1^
Glucuronide of C10H18O2 (9)^2^	0.011 (0.005, 0.017)^1^	0.011 (0.005, 0.017)^1^	0.012 (0.006, 0.019)^1^
X-25442	0.011 (0.005, 0.017)^1^	0.013 (0.007, 0.019)^1^	0.012 (0.006, 0.019)^1^
X-17825	0.009 (0.003, 0.015)^1^	0.011 (0.004, 0.017)^1^	0.011 (0.005, 0.017)^1^
X-24345	0.010 (0.004, 0.016)^1^	0.012 (0.006, 0.018)^1^	0.012 (0.005, 0.018)^1^
X-17358	0.011 (0.005, 0.017)^1^	0.010 (0.004, 0.017)^1^	0.011 (0.005, 0.018)^1^
1,6-anhydroglucose	0.010 (0.004, 0.016)^1^	0.011 (0.005, 0.017)^1^	0.011 (0.004, 0.018)^1^
3-indoxyl sulfate	0.003 (–0.003, 0.009)	0.008 (0.002, 0.014)	0.010 (0.004, 0.016)^1^
Glycerophosphorylcholine (GPC)	0.005 (–0.001, 0.011)	0.009 (0.002, 0.015)	0.011 (0.004, 0.017)^1^
Glycolate (hydroxyacetate)	0.012 (0.006, 0.018)^1^	0.010 (0.004, 0.016)^1^	0.010 (0.004, 0.016)^1^
6-Bromotryptophan	0.011 (0.005, 0.017)^1^	0.010 (0.004, 0.016)^1^	0.010 (0.004, 0.016)^1^

Abbreviations: *β*, regression estimate; CI, confidence interval; UPF, ultraprocessed food.

Model 1: Unadjusted.

Model 2: Adjusted for age, sex, BMI, and energy intake.

Model 3: Adjustments in model 2 and physical activity, alcohol and smoking status.

Each model was run independently (i.e., separately not based on statistical significance in previous model), overall significance was based on model 3.

1 Statistically significant results (false discovery rate-adjusted *q* value < 0.05). Only positive associations are summarized; full results are given in Supplemental Table 2.

2 Indicates a compound that has not been confirmed based on authentic chemical standard, but Metabolon are confident in its identity. The structural identities of “X-” followed by a number (e.g., X-17679) are unknown.

Among the known metabolites, higher UPF intake was associated with higher concentrations of indoxyl glucuronide *β* = 0.013 [95% confidence intervals (CIs): 0.007, 0.019] and other partially characterized glucuronides: glucuronide of C10H18O2 (1), *β* = 0.015 (0.008, 0.021); glucuronide of C10H14O2 (2), *β* = 0.015 (0.008, 0.021); glucuronide of C10H18O2 (7), *β* = 0.014 (0.007, 0.021), glucuronide of C10H18O2 (8), *β* = 0.014 (0.007, 0.020). Other metabolites were N, N-dimethylalanine, *β* = 0.013 (0.007, 0.019); 1-methylhistamine, *β* = 0.012 (0.006, 0.018); 3-Hydroxy-N6,N6,N6-trimethyl-L-lysine, *β* = 0.012 (0.006, 0.018); glycerophosphorylcholine, *β* = 0.011 (0.004, 0.017); 1,6-anhydroglucose, *β* = 0.011 (0.004, 0.018); 3-indoxyl sulfate, *β* = 0.010 (0.004, 0.016); glycolate, *β* = 0.010 (0.004, 0.016); and 6-bromotryptophan, *β* = 0.010 (0.004, 0.016) (Table 2).

There were also structurally unknown metabolites whose concentrations positively correlated with UPF intake, namely X-17679, *β* = 0.027 (0.021, 0.033); X-19497, *β* = 0.016 (0.010, 0.022); X-11478, *β* = 0.014 (0.07, 0.020), among others (Table 2).

### Associations between UPF intake and plasma metabolites

In young adult plasma samples, UPF intake was associated with 6 metabolites in our fully adjusted model after corrections for multiple testing (Table 3). Of these, UPF intake was associated with elevated concentrations of 4-hydroxyglutamate, *β* = 0.021 (0.011, 0.031) and 2 structurally unknown metabolites, X-11372, *β* = 0.019 (0.009, 0.030) and X-24951, *β* = 0.019 (0.009, 0.030).

**TABLE 3:** Regression estimates of the associations between UPF intake and plasma metabolites in young adults (*N* = 195).

Metabolite	Model 1, *β* (95% CI)	Model 2, *β* (95% CI)	Model 3, *β* (95% CI)
Homostachydrine^1^	–0.021 (–0.031, –0.011)^2^	–0.025 (–0.035, –0.015)^2^	–0.024 (–0.034, –0.013)^2^
4-Hydroxyglutamate	0.017 (0.007, 0.028)	0.023 (0.013, 0.033)^2^	0.021 (0.011, 0.031)^2^
3-CMPFP	–0.019 (–0.029, -0.009)	–0.020 (–0.031, –0.010)^2^	–0.020 (–0.031, –0.009)^2^
X-11372	0.020 (0.009, 0.030)	0.018 (0.008, 0.028)^2^	0.019 (0.009, 0.030)^2^
X-23639	–0.015 (–0.025, –0.004)	–0.020 (–0.030, –0.009)^2^	–0.020 (–0.031, –0.010)^2^
X-24951	0.018 (0.008, 0.028)	0.019 (0.009, 0.029)^2^	0.019 (0.009, 0.030)^2^

Abbreviations: *β*, regression estimate; 3-CMPFP, 3-carboxy-4-methyl-5-pentyl-2-furanpropionate; CI, confidence interval; UPF, ultraprocessed food.

Model 1: Unadjusted.

Model 2: Adjusted for age, sex, BMI, energy intake, number of dietary assessments, and time difference between dietary assessment and blood draw.

Model 3: Adjustments in model 2 and physical activity, alcohol, and smoking status.

1 Indicates a compound that has not been confirmed based on authentic chemical standard, but Metabolon are confident in its identity. The structural identities of “X-” followed by a number (e.g., X-11372) are unknown.

2 Statistically significant results (false discovery rate-adjusted *q* value <0.05).

### Associations between UPF intake and urine and plasma MPs

In adolescent urine samples, 25 MPs, explaining 61.7% of urine metabolite variation, were analyzed in relation to UPF intake (Supplemental Table 3). Four MPs (MP7, MP9, MP10, and MP18) were associated with UPF intake in the minimally adjusted model (Table 4). Two of these associations were independent of lifestyle factors and multiple testing correction: MP9, *β* = 0.042 (0.014, 0.070) and MP7, *β* = –0.063 (–0.092, –0.034). In brief, the MP9, consisting of *n* = 214 metabolites, was dominated by metabolites in the xenobiotic super pathway (63 metabolite of subclasses: food components, xanthine metabolism, chemicals and drugs, and benzoate metabolism), 64 structurally unknown metabolites, 40 amino acids, 16 lipids, 11 partially characterized molecules (particularly, glucuronides of C8H14O2, C8H14O2, C8H16O2, C10H18O2, C12H22O4, C12H22O3, and C14H26O4), and the rest were spread across nucleotides, cofactors and vitamins, energy, and peptides. Therefore, on the basis of known metabolic pathways, we named this urinary pattern “xenobiotics and amino acids” MP. The MP7, consisting of *n* = 281 metabolites, was dominated by unknown metabolites (*n* = 74), amino acids (*n* = 70), lipids (*n* = 51), and xenobiotics (*n* = 41) among other metabolite classes. We named this urinary pattern, “amino acids, lipids, and xenobiotics” MP.

**TABLE 4:** Regression estimates of associations between UPF intake and urine and plasma metabolite patterns.

Analytic sample	MP	Model 1, *β* (95% CI)	Model 2, *β* (95% CI)	Model 3, *β* (95% CI)
Adolescent urine	MP7	–0.062 (–0.090, –0.034)^1^	–0.064 (–0.092, –0.036)^1^	–0.063 (–0.092, –0.034)^1^
	MP9	0.046 (0.20, 0.072)^1^	0.046 (0.019, 0.073)^1^	0.042 (0.014, 0.070)^1^
	MP10	0.022 (–0.003, 0.047)	0.036 (0.011, 0.061)^1^	0.029 (0.003, 0.055)
	MP18	–0.020 (–0.042, 0.002)	–0.032 (–0.055, –0.010)^1^	–0.032 (–0.056, –0.008)
Young adult plasma				
	MP1	–0.021 (–0.100, 0.058)	–0.081 (–0.134, –0.029)^1^	–0.064(–0.119, –0.010)
	MP6	0.047 (0.005, 0.088)	0.063 (0.023, 0.104)^1^	0.049 (0.008, 0.091)
	MP8	0.065 (0.026, 0.105)^1^	0.072 (0.031, 0.113)^1^	0.074 (0.031, 0.117)^1^
	MP17	–0.040 (–0.071, –0.010)	–0.052 (–0.083, –0.021)^1^	–0.038 (–0.070, –0.007)

Abbreviations: *β*, regression estimate; CI, confidence interval; MP, metabolite pattern; UPF, ultraprocessed food.

Model 1: Unadjusted.

Model 2: Adolescent urine – adjusted for age, sex, BMI, and energy intake.

Model 2: Young adult plasma – adjusted for age, sex, BMI, energy intake, number of dietary assessments, and time difference between dietary assessment and blood draw.

Model 3: Adjustments in model 2 and physical activity, alcohol and smoking status.

The MPs were analyzed and labeled separately for urine and plasma samples as MP1 to MPn.

1 Statistical significance (false discovery rate-adjusted *q* value <0.05). Full results are given in Supplemental Tables 3 and 6.

We compared the 2 urinary MPs 9 and 7 and found 46 common metabolites, mostly showing opposite direction of PCA loadings, which also possibly reflects the results observed in multivariable linear regression models (i.e., UPF’s positive association with MP9 and inverse association with MP7). Of these common metabolites, 17 represented xenobiotic metabolism (subclasses: food component, xanthine metabolism, and chemicals) and 13 amino acids (primarily involved in glycine, serine, and threonine; tryptophan; and alanine and aspartate metabolism) and other unknown metabolites and pathways. Extended tables of metabolites represented in MP9 and MP7 are provided in Supplemental Tables 4 and 5, with the top metabolites contributing to the variation and their loadings summarized in Supplemental Figures 3 and 4, respectively.

In young adult plasma samples, 19 MPs explaining 55.0% of the plasma metabolite variation were analyzed with the UPF intake (Supplemental Table 6). Four MPs (MP1, MP6, MP8, and MP17) were associated with UPF intake in the minimally adjusted model (Table 4). Of these, only MP8 was associated with UPF intake in our fully adjusted model after corrections for multiple testing, *β* = 0.074 (0.031, 0.117). This MP8 had *n* = 216 metabolites, dominated by lipids (*n* = 86), unknown metabolites (*n* = 45), xenobiotics (*n* = 39) amino acids (*n* = 28), and the rest spread across cofactors and vitamins, peptides, and partially characterized molecules. Similarly, on the basis of the known biochemical pathways, we named this pattern “lipids, xenobiotics, and amino acids” MP. An extended table of this MP is provided in Supplemental Table 7 and its top metabolites and weights in Supplemental Figure 5.

Lastly, we compared the similarity of the UPF-positively associated MPs across biological matrices (urine and plasma, i.e., urinary MP9 reflecting short-term intake and plasma MP8, reflecting repeated, long-term intake). We found 29 common metabolites, mostly xenobiotics (*n* =15) of subclasses food components, benzoate, xanthine, and drug/chemical pathways, and the rest were mostly amino acids and lipids.

### Secondary and sensitivity analyses

In adolescent urine samples, most metabolites generally showed comparable results for both absolute and energy-based UPF variables, with few exceptions. For example, saccharin, a common noncaloric ingredient in sweetened beverages, was not statistically significant even before correcting for multiple statistical tests for the energy-based UPF, *β* = 0.005 (–0.003, 0.012) but was significant in absolute UPF intake, *β* = 0.0002 (0.0000, 0.0004) (Supplemental Table 8). The UPF-associated urinary MP9 was statistically significant with absolute and energy-based UPF, whereas the MP7 was statistically significant with energy-based UPF but not with the absolute UPF model (Supplemental Table 9).

In young adult plasma samples, most associations observed with the weight-based ratio were also replicated using absolute UPF intake and energy-based UPF (Supplemental Table 10). The UPF-related plasma MP8 was statistically significant with all UPF variable specifications (Supplemental Table 11).

Regarding the correlation between UPF and UMF intakes, we found a strong negative correlation of these intakes in both adolescent urine, *r* = –0.88 (–0.90, –0.86), and young adult plasma, *r* = –0.95 (–0.96, –0.95), samples.

In our sensitivity analyses on potentially implausible intakes, excluding possibly underreported intakes showed comparable results to those obtained from the main analysis using the entire analytic samples regardless of the UPF variable specification, urinary patterns (Supplemental Table 12) and plasma patterns (Supplemental Table 13).

## Discussion

Using untargeted metabolomics, we investigated cross-sectional associations of UPF intake with urine metabolites and associations of repeatedly assessed UPF intake with plasma metabolites in a cohort of free-living adolescents and young adults. Our results suggest that the intake of UPF is reflected in the urine and plasma metabolome, through diverse biochemical pathways such as xenobiotics, amino acids, and lipids pathways, including alterations of microbiome-derived and other endogenous metabolites.

Noteworthy single-metabolite associations in urine included indoxyl glucuronide and several other partially characterized glucuronides. Glucuronidation is a major pathway for detoxification and elimination of exogenous substances, predominantly drugs, chemicals, dietary substances, and endogenous compounds such as hormones [48]. A recent controlled feeding trial also reported UPF-related changes in indoxyl glucuronide concentrations and various glucuronides of C10H18O2 in 24-h urine [28]. Similarly, we previously reported these associations (glucuronide of C10H14O2, glucuronide of C10H18O2) with sweetened beverages in 24-h urine samples [33], the food group with highest contribution to the UPF intake in our study. The specific mechanisms for dietary-related glucuronidation are not entirely defined, but the gut microbiome plays a key role in modulating microbial transformation of dietary substrates and glucuronide levels [48] and biosynthesis of microbial metabolites [49].

Indeed, 3-indoxyl sulfate, a protein-bound uremic toxin, and 6-bromotryptophan, both positively associated with UPF intake are microbiome-derived tryptophan metabolites. This association of UPF intake with indoxyl sulfate was also observed in adults [28]. These metabolites may be important because of the suggested roles of the UPFs and western-style diets in inducing gut microbial dysbiosis [[50], [51], [52]], and the associations of microbiome-related metabolites with health outcomes. In other studies, higher indoxyl sulfate concentrations were positively linked with various health problems: psychic anxiety [53], cognitive impairment [54], and neuroinflammation and oxidative stress [55].

Our finding of a positive association of UPFs with 1-methylhistamine is also consistent with a previous study on gut metabolites and microbial compositions, which observed that children on a western-style diet had elevated concentrations of 1-methylhistamine compared with those on a Mediterranean-style diet [56]. The mechanisms for elevated levels of 1-methylhistamine with UPF intake are unclear and could reflect histamine release as an allergic response [57], food histamine intolerance [58], or even microbiological contamination of certain foods [59].

The N,N-dimethylalanine, positively associated with the UPF in this study and similarly with sweetened beverages in our previous study [33], may be important in various biological processes. For instance, the urinary N,N-dimethylalanine was inversely associated with 3 measures of adiposity in adolescents [33], and lower concentrations of plasma N,N-dimethylalanine were observed in adults with type 2 diabetes in another study [60]. There is, however, limited literature on this metabolite.

Consistent with the study by O’Connor et al. [28], we observed that higher UPF intakes were associated with lower concentrations of metabolites known to reflect minimally processed or certain whole foods. Our single-metabolite models showed that higher UPF intake was linked to lower levels of hydroquinone sulfate, a marker of pear intake [61]; dopamine 3-O-sulfate, a marker for banana intake [62,63]; 2-acetamidophenol sulfate (HPAA sulfate), linked to whole grains [64]; 3,5-dihydroxybenzoic acid, also associated with intake of whole-grain cereals [47,65]; allantoin, a purine derivative found in cow milk [66]; caffeic acid sulfate, a polyphenol abundant in coffee beans [62]; ferulic acid 4-sulfate, a metabolite of ferulic and caffeic acid found in fruits, whole-grain cereals, and coffee [67] and 3-methyladipate, a metabolite of phytanic acid breakdown, found in meat, dairy fat, and fatty fish [68]. Taken together, these data suggest lower intake of these foods in diets rich in UPF.

The single-metabolite associations with UPFs were also captured in the urinary “xenobiotics and amino acids” pattern, MP9; whose composition includes nutrient- and nonnutrient-related metabolites. Many of these metabolites were reported in a previous feeding study [28]. Metabolites of xanthine metabolism, for example, caffeine and theobromine were also related to UPF intake in another study [10], whereas concentrations of theobromine, 7-methylxanthine, and 3-methylxanthine were elevated with UPF intake [28]. In our previous study including the same participants [33], we observed that these caffeine-related metabolites more likely reflected caffeine in the sweetened beverages independent of other dietary sources of caffeine. Theobromine is a well-known component of cocoa and present in chocolate and other cocoa containing foods. Thus, considering the main contributing foods to the UPF intake in our study, these metabolites and their variation (i.e., direction of their PCA loadings) relative to those known to reflect whole foods, make this pattern compellingly relevant to the UPF.

Furthermore, one of the distinctive characteristics of UPFs, according to the NOVA system, is the use of industrial food additives such as flavoring and preservative agents. In MP9, metabolites reflecting these include vanillate (4-hydroxy-3-methoxybenzoate), a widely used vanilla food flavor [69] and naringenin 7 glucuronide, metabolite of naringenin, an industrial flavoring agent extracted from grapefruit [70]. We, however, note that other food sources of naringenin exist, such as citrus fruits and fruit juices [71]. In addition, various forms of benzoates (e.g., sodium benzoate) are commonly used as preservatives in packaged foods [72]. Metabolites of benzoate metabolism pathway in the UPF-related MP9 were 3-methyl catechol sulfate, 4-ethylcatechol sulfate, 2-ethylphenylsulfate, and o-cresol.

In relation to long-term UPF intake and plasma metabolites, we observed far fewer associations compared with urine using the single-metabolite modeling approach. This might suggest generally weak individual plasma metabolite associations with UPF intake, with only a few remaining significant after correction for multiple testing. These differences could also reflect the biological characteristics of the sample matrices. Urine is a more reliable matrix for investigating short-term response to dietary intakes and detoxification pathways, whereas blood matrix more reliably reflects endogenous metabolism of food from the gut to the liver and blood [20]. An interesting result in our single-metabolite models was the association of higher UPF intake with elevated concentration levels of 4-hydroxyglutamate (4-hydroxy-L-glutamic acid). This association was recently observed in spot urine, 24-h urine, and plasma samples in a controlled feeding study by O’Connor et al. [28]. Given that 4-hydroxyglutamate is implicated in metabolic syndrome [73] and pre-eclampsia [74,75], its mechanistic link with the UPFs could be an interesting research target regarding diet–health relationships.

The UPF-plasma MP8 largely reflected lipid metabolites. Differences and variation of dietary modulation of urine and blood metabolome are anticipated [20], and some differences in our results may also reflect some of the long-term changes associated with UPF intake. Importantly, the common metabolites across the urine and plasma MPs positively linked to UPF were mostly involved in xenobiotic metabolism of food-related components, xanthine metabolism, benzoates, sulfites, and metabolites of potential contaminants or exposures to chemicals or drugs. Therefore, although unsupervised machine learning methods such as the PCA are exploratory and multiple biological interpretations may exist for observed exposure associations, collectively, the common biochemical pathways and their joint combinations as captured in these urine and plasma MPs reinforce their potential relevance to UPF intake.

In summary, our study provides further evidence and insights into the growing concern of the potential health effects of UPFs. Our data showed a strong negative correlation between intakes of UPF and UMF, suggesting that UPFs displace whole foods that should be the basis of the diet according to the NOVA system. These results are consistent with established literature and long-held perspectives on public health nutrition [2,4]. In line, our study extends this evidence to molecular level, as shown in our explorative single-metabolite models and MPs. We also note that the UPFs were associated with changes in gut microbiota metabolites (e.g., indoxyl sulfate and 6-bromotryptophan). Mechanisms for gut bacteria–xenobiotics interplay are well described [76], and western-style diets [51,52,77] or food additives themselves [50] are suggested to induce gut microbiome dysbiosis. Thus, our results suggest multiple ways through which regular intakes of UPFs may influence health—from nutritional displacement to alterations of the gut microbiota composition, and possibly other unintended but undetermined effects of industrial food additives and formulations. Some of these metabolites, which seem metabolically or physiologically important, could be putative links between the consumption of UPFs and health status.

This study has several strengths. The DONALD study design allows for regular, repeated dietary assessments on the same individuals that have enabled assessment of long-term UPF intake. The DONALD’s 3d-WDRs, compared with other methods such as food-frequency questionnaires, collects dietary data at the food item level rather than at food group level, enabling more accurate assigning of their NOVA groups. Using urine and blood—the most popular biological matrices—and their complementary nature enabled us to investigate short-term and long-term metabolomics profiles of UPF intake. This approach has the potential to provide more insights on potentially transient as well as sustained metabolomics alterations related to dietary intakes. Besides, by combining single-metabolite models with the MP approaches, we identified individual metabolites as well as broader MPs that may mechanistically reflect the UPF-associated perturbations of the metabolome. We opted for nontargeted metabolomics approaches given that targeting single or multiple selected pathways might not optimally reflect the complex UPF influence on the metabolome.

We acknowledge several limitations in this study. Self-reported dietary assessments are subject to random and systematic errors. We performed sensitivity analysis on potential implausible dietary reporting, although these checks are based on energy intakes and only identifies potential underreporting or over-reporting. Another challenge relates to the uncertainty in classification of some multi-ingredient foods into processed (NOVA-3) or the UPFs (NOVA-4). Despite reaching consensus for food groupings based on multiple guidelines, some discrepancies may still exist. Furthermore, some of the metabolites consistently associated with UPF intakes in single-metabolite models and MPs were of unknown structural identities, limiting our understanding of their biological functions. Lastly, the DONALD cohort consists of a largely homogeneous, urban population with a higher socioeconomic status than the general German population [32]. This homogeneity could influence their food choices and dietary habits. Nonetheless, given that this study replicated findings of other studies from different populations and regions, suggests reasonable generalizability of our findings.

In conclusion, we identified individual metabolites and MPs that reflect UPF intake in urine and plasma samples of adolescents and young adults. These findings add to the growing literature on complementary assessment of UPF intake and reports some of the underlying biological mechanisms through which these foods may affect metabolism and health. Besides, the extensive processing of food resulting in UPFs has generated considerable clinical and public health interest and become mired in controversy. Our data suggest that the complex and heterogeneous nature of UPFs may be gleaned from the metabolome.

## Author contributions

The authors’ responsibilities were as follows – UN, AF, SM: conceptualization and research design; AB, CAC, MEB, UA, SM: compiled dietary data; PK-R: conducted research; SM: analyzed data and wrote the first draft; MS: statistical supervision; SM, UN: had primary responsibility for final content, and all authors: read and approved the final manuscript.

## Funding

The authors acknowledge funding from the German Research Foundation (DFG 406710821) and the Agence Nationale de la Recherche; the Diet–Body–Brain (DietBB), the Competence Cluster in Nutrition Research (Federal Ministry of Education and Research, FKZ:01EA1410A); and the PerMiCCion project (Federal Ministry of Education and Research, Grant 100554612).

## Data availability

Data described in the manuscript are not publicly available due to ethical restrictions, but are available upon reasonable request, pending application and approval. Data requests to be addressed to Prof. Ute Nöthlings via epi@uni-bonn.de.

## Conflict of interest

The authors report no conflicts of interest. Where authors are identified as personnel of the International Agency for Research on Cancer/WHO, the authors alone are responsible for the views expressed in this article and they do not necessarily represent the decisions, policy, or views of the International Agency for Research on Cancer/WHO.
