# Supplementary material for: Association of Ultraprocessed Foods Intake with Untargeted Metabolomics Profiles in Adolescents and Young Adults in the DONALD Cohort Study
Source: J Nutr. 2024 Sep 25;154(11):3255–65. doi: 10.1016/j.tjnut.2024.09.023 (PMC11600117; doi:10.1016/j.tjnut.2024.09.023)
Supplement: multimedia component 1 [file mmc1.pdf]

Supplemental materials to

Muli et al. Association of ultra-processed foods intake with untargeted metabolomics profiles in adolescents and young adults in the DONALD cohort study.

Supplemental Figure 1. Study population

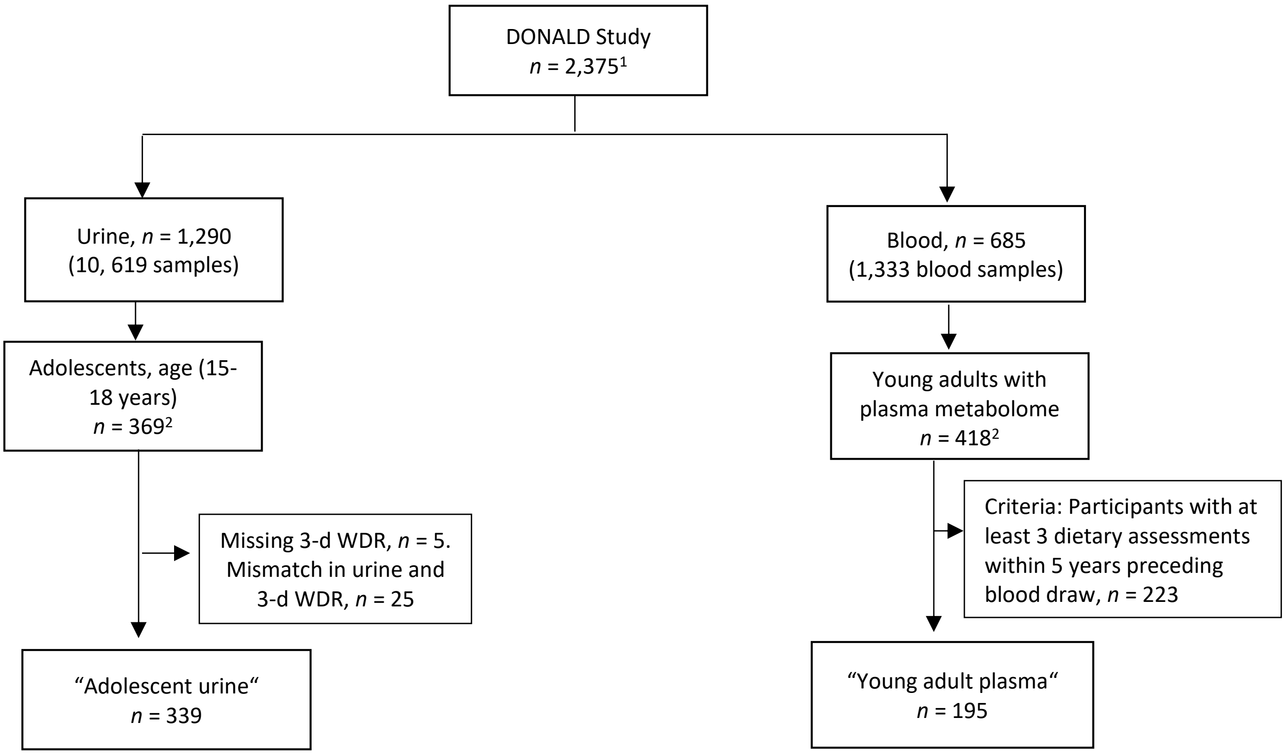

Supplemental Figure 1. Flowchart of the DONALD study samples included in the present analysis. [1]participants recruited between 1985 and December 2022. [2] these two samples were randomly selected among eligible urine and blood samples. There was an overlap of *n* = 139 participants across the two analytic samples.

Supplemental Table 1. NOVA classification system (modified according to Monteiro et al. 2019)[1]

NOVA-1: Unprocessed and minimally processed foods	Fresh, squeezed, chilled, frozen, or dried fruit and leafy and root vegetables; grains (parboiled or white rice, corn cob or kernel, wheat berry or grain); legumes (beans, lentils, and chickpeas); starchy roots and tubers (potatoes, sweet potatoes and cassava); fungi (fresh or dried mushrooms); meat, poultry, fish and seafood, fresh, powdered, chilled or frozen eggs; fresh, powdered or pasteurized milk; fresh or pasteurized fruit or vegetable juices (with no added sugar, sweeteners or flavours); grits, flakes or flour made from corn, wheat, oats, or cassava; tree and ground nuts and other oily seeds (with no added salt or sugar); herbs and spices (thyme, oregano, mint, pepper, cloves, cinnamon); fresh or pasteurized plain yoghurt; tea, coffee, drinking water
NOVA-2: Processed culinary ingredients	Vegetable oils crushed from seeds, nuts or fruit (notably olives); butter and lard obtained from milk and pork; sugar and molasses obtained from cane or beet; honey extracted from combs and syrup from maple trees; starches extracted from corn and other plants; vegetable oils with added antioxidants; salt mined or from seawater, and table salt with added drying agents
NOVA-3: Processed foods	Canned or bottled vegetables and legumes in brine; salted or sugared nuts and seeds; salted, dried, cured, or smoked meats and fish; canned fish; fruit in syrup; freshly made unpackaged breads and cheeses
**NOVA-4: Ultra-processed foods**	carbonated soft drinks; sweet or savory packaged snacks; chocolate, candies (confectionery); ice-cream; mass-produced packaged breads and buns; margarines and other spreads; cookies (biscuits), pastries, cakes, and cake mixes; breakfast 'cereals', 'cereal' and 'energy' bars; 'energy' drinks; milk drinks, 'fruit' yoghurts and 'fruit' drinks; 'cocoa' drinks; 'instant' sauces; pies and pasta and pizza dishes; poultry and fish 'nuggets' and 'sticks', sausages, burgers, hot dogs, and other reconstituted meat products; and powdered and packaged 'instant' soups, noodles and desserts; infant formulas, follow-on milks, other baby products; 'health' and 'slimming' products (meal replacement shakes and powders)
	**Examples of food items in NOVA-4/UPF Subgroups[2]**
**Soft drinks**	Sweetened fruit juice drinks, fruit nectars, lemonades, cola drinks, fizzy drinks, iced tea, malt beer, draught soda, fruity punch
**Convenient, ready-to-heat/eat foods**	Instant soups, ready to eat instant sauces, gravies, instant dressings, tomato (e.g., ketchup, spicy fruit sauces) /vegetable sauces, non-vegetable based sauces, any other sauce (warm or cold), convenience food based on grain (e.g., Pizza, ravioli with sauce, cheese spaetzle, lasagne, filled wraps, onion tart, potato salad, pasta salad, potato soup, Djuvec rice, spring rolls, etc), convenience foods based on vegetables/pulses (e.g., creamed spinach, buttered vegetables, falafel, lentil and pea stew, vegetable burgers,

	salads, apple and red cabbage, sauerkraut, spicy fruit dishes e.g. mango curry), convenience foods based on meat/fish, non-milk desserts
**Yogurts and diary based drinks**	All processed dairy-based drinks, fermented and unfermented.
**Sweets, chocolates, ice cream**	Sugar, syrups, thick juice, juice concentrates, sweeteners and sugar substitutes, stevia; sweet parfait e.g. jam, honey, hazelnut spread (not pure nut butter), pear syrup, sugar beet syrup; candy e.g. wine gums, drops, chewing gum, chewy candy, licorice, dextrose, edible paper; chocolate, bars including muesli bars, fruit bars, chocolate sprinkles, chocolates (including Raffaello), sweets/nuts/popcorn/puffed rice with chocolate coating; water ice, milk ice, fruit ice
**Processed meats and sausages**	Sausages, cold cuts, and other processed meat dishes
**Cereal products, breads**	Ready-to-eat cereals, muesli, mass-produced packaged breads, buns and other bread products (e.g., Raisin bread, rolls, crispbread, savory croissants, etc.)
**Processed fruit products**	Frozen mixes with other ingredients such as alcohol, juice and added sugar, canned fruit and fruit purée, dried fruit, fruit juices and fruit and vegetable juices (if at least 50 % fruit), juice spritzer (still/sparkling water or tea with juice), smoothies and juices with added milk, nuts, seeds, trail mix (also with dried fruit, ≥50% nuts), roasted/salted nuts, peanuts, roasted almonds, pure nut butter
**Biscuits, cakes, pastries, savoury**	Firm, dry biscuits e.g. cookies, rusks, gluten-free products, rice cakes; cakes and (soft) pastries e.g. yeast rolls, muffins, donuts, fresh egg waffles, Magdalenas, honey cakes, baked cake bases, gluten-free cakes; savoury, salty snacks e.g. potato chips, pretzel sticks, grissini, crackers, bread potato chips, rice cakes, spelt sticks
**Cheese, other dairy products**	Fresh cheese e.g. curd cheese desserts, tzatziki, cottage cheese, tiramisu; (semi) hard cheese e.g. Gouda, Edam cheese; soft cheese e.g. Camembert, blue cheese, feta, mozzarella, grilled cheese; processed cheese (for spreading and as slices), dairy powder e.g. whole milk powder, whey powder, yogurt powder, curd powder, milk protein concentrate, cream powder
**Potato products**	Fried or mashed potatoes, french fries, potato dumplings, fried grated potatoes, gnocchi, potato pancakes, potato noodles ‘Schupfnudeln’ (without sauce), powder for dumplings or puree
**Alcoholic beverages**	All alcoholic beverages, non-alcoholic beer, non-alcoholic sparkling wine, shandy

**Processed fish products**	Salads, canned fish, fish sticks, smoked fish, seafood, surimi, caviar, pre-marinated fish
**Margarine, spreads**	Processed animal fat e.g., salted and unsalted butter, herb butter, lard, tallow, salmon oil, cod liver oil, non-smoked back bacon, margarine, vegetable oil, vegetable fat e.g. plant creams, coconut oil, Biskin
**Noodles**	Ready-made doughs, raw, cooked or fresh pasta (tortellini, ravioli, spaetzle, bread dumplings, rice noodles)
**Instant beverages, teas**	For water and milk, coffee, bagged and instant teas
**Vegan/vegetarian substitutes**	Vegan milk substitute e.g. cereal milks, soy milks, soy yogurt, vegan desserts, coconut milk; vegetarian/vegan spread, vegetarian/vegan meat/fish/egg substitute e.g. tofu, soy sausages, soy granules, cereal patty mix, pâtés

[1]Monteiro CA, Cannon G, Levy RB, Moubarac JC, Louzada ML, Rauber F, et al. Ultra-processed foods: what they are and how to identify them. Public Health Nutr. 22 (2019), 936–941.

[2] These food items were included only if they met the criteria for the NOVA-4 category.

A

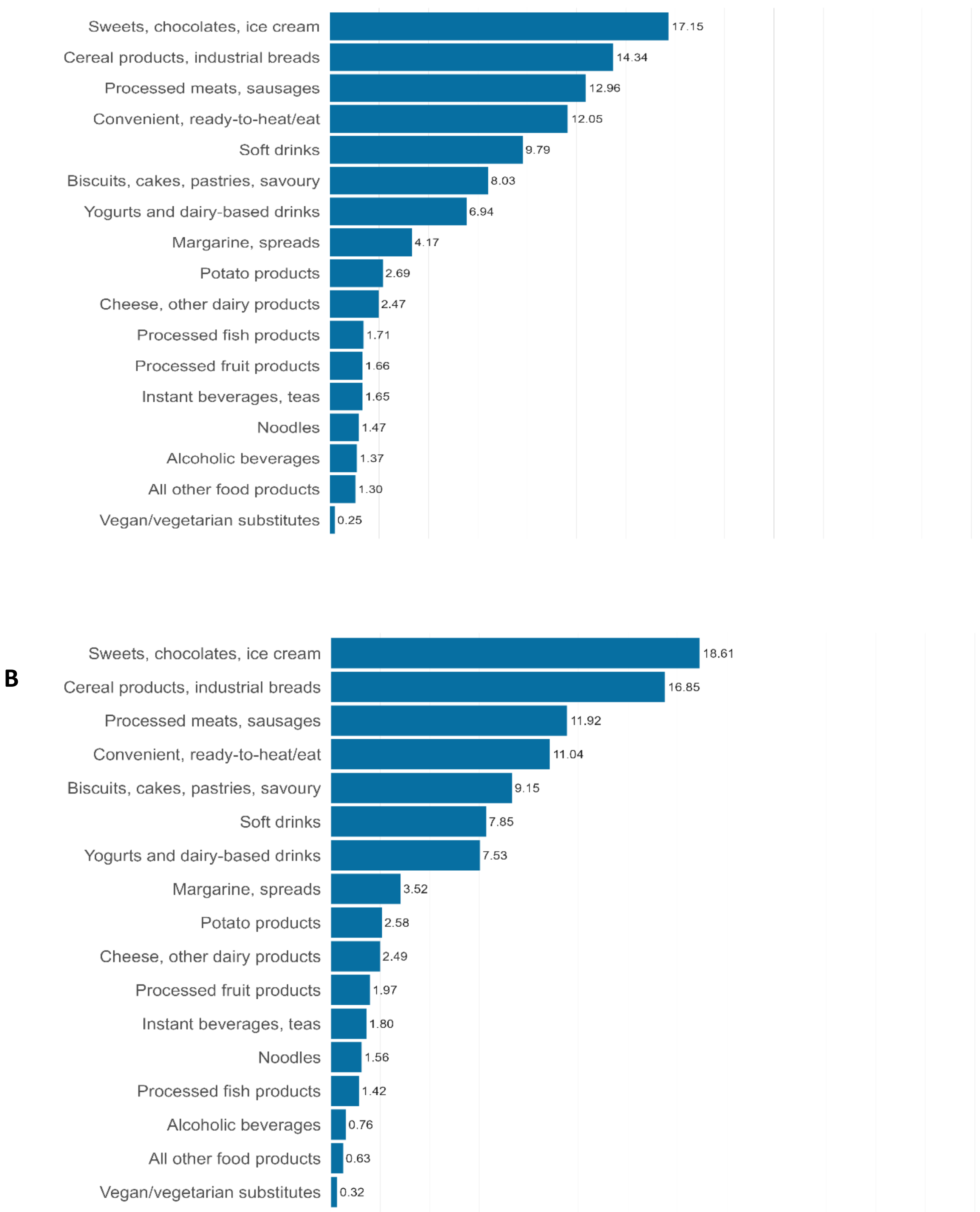

Supplemental Figure 2. Mean energy contributions of various foods groups to the total UPF energy intake (%) in (**A**) Adolescent urine and (**B)** Young adult plasma analytic samples

Metabolon's Metabolomics Analysis Report

Metabolon Platform

Sample Accessioning: Following receipt, samples were inventoried and immediately stored at -80°C. Each sample received was accessioned into the Metabolon LIMS system and was assigned by the LIMS a unique identifier that was associated with the original source identifier only. This identifier was used to track all sample handling, tasks, results, etc. The samples (and all derived aliquots) were tracked by the LIMS system. All portions of any sample were automatically assigned their own unique identifiers by the LIMS when a new task was created; the relationship of these samples was also tracked. All samples were maintained at -80°C until processed.

Sample Preparation: Samples were prepared using the automated MicroLab STAR® system from Hamilton Company. Several recovery standards were added prior to the first step in the extraction process for QC purposes. To remove protein, dissociate small molecules bound to protein or trapped in the precipitated protein matrix, and to recover chemically diverse metabolites, proteins were precipitated with methanol under vigorous shaking for 2 min (Glen Mills GenoGrinder 2000) followed by centrifugation. The resulting extract was divided into five fractions: two for analysis by two separate reverse phase (RP)/UPLC-MS/MS methods with positive ion mode electrospray ionization (ESI), one for analysis by RP/UPLC-MS/MS with negative ion mode ESI, one for analysis by HILIC/UPLC-MS/MS with negative ion mode ESI, and one sample was reserved for backup. Samples were placed briefly on a TurboVap® (Zymark) to remove the organic solvent. The sample extracts were stored overnight under nitrogen before preparation for analysis.

QA/QC: Several types of controls were analyzed in concert with the experimental samples: a pooled matrix sample generated by taking a small volume of each experimental sample (or alternatively, use of a pool of well-characterized human plasma) served as a technical replicate throughout the data set; extracted water samples served as process blanks; and a cocktail of QC standards that were carefully chosen not to interfere with the measurement of endogenous compounds were spiked into every analyzed sample, allowed instrument performance monitoring and aided chromatographic alignment. Instrument variability was determined by calculating the median relative standard deviation (RSD) for the standards that were added to each sample prior to injection into the mass spectrometers. Overall process variability was determined by calculating the median RSD for all endogenous metabolites (i.e., non-instrument standards) present in 100% of the pooled matrix samples. Experimental samples were randomized across the platform run with QC samples spaced evenly among the injections

Ultrahigh Performance Liquid Chromatography-Tandem Mass Spectroscopy (UPLC-MS/MS): All methods utilized a Waters ACQUITY ultra-performance liquid chromatography (UPLC) and a Thermo Scientific Q-Exactive high resolution/accurate mass spectrometer interfaced with a heated electrospray ionization (HESI-II) source and Orbitrap mass analyzer operated at 35,000 mass resolution. The sample extract was dried then reconstituted in solvents compatible to each of the four methods. Each reconstitution solvent contained a series of standards at fixed concentrations to ensure injection and chromatographic consistency. One aliquot was analyzed using acidic positive ion conditions, chromatographically optimized for more hydrophilic compounds. In this method, the extract was gradient eluted from a C18 column (Waters UPLC BEH C18-2.1x100 mm, 1.7 µm) using water and methanol, containing 0.05% perfluoropentanoic acid (PFPA) and 0.1% formic acid (FA). Another aliquot was also analyzed using acidic positive ion conditions, however it was chromatographically optimized for more hydrophobic compounds. In this method, the extract was gradient eluted from the same afore mentioned C18 column using methanol, acetonitrile, water, 0.05% PFPA and 0.01% FA and was operated at an overall higher organic content. Another aliquot was analyzed using basic negative ion optimized conditions using a separate dedicated C18 column. The basic extracts were gradient eluted from the column using methanol and water, however with 6.5mM Ammonium Bicarbonate at pH 8. The fourth aliquot was analyzed via negative ionization following elution from a HILIC column (Waters UPLC BEH Amide 2.1x150 mm, 1.7 µm) using a gradient consisting of water and acetonitrile with 10mM Ammonium Formate, pH 10.8. The MS analysis alternated between MS and data-dependent MS^n scans using dynamic exclusion. The scan range varied slighted between methods but covered 70-1000 m/z. Raw data files are archived and extracted as described below.

Bioinformatics: The informatics system consisted of four major components, the Laboratory Information Management System (LIMS), the data extraction and peak-identification software, data processing tools for QC and compound identification, and a collection of information interpretation and visualization tools for use by data

analysts. The hardware and software foundations for these informatics components were the LAN backbone, and a database server running Oracle 10.2.0.1 Enterprise Edition.

LIMS: The purpose of the Metabolon LIMS system was to enable fully auditable laboratory automation through a secure, easy to use, and highly specialized system. The scope of the Metabolon LIMS system encompasses sample accessioning, sample preparation and instrumental analysis and reporting and advanced data analysis. All of the subsequent software systems are grounded in the LIMS data structures. It has been modified to leverage and interface with the in-house information extraction and data visualization systems, as well as third party instrumentation and data analysis software.

Data Extraction and Compound Identification: Raw data was extracted, peak-identified and QC processed using Metabolon's hardware and software. These systems are built on a web-service platform utilizing Microsoft's .NET technologies, which run on high-performance application servers and fiber-channel storage arrays in clusters to provide active failover and load-balancing. Compounds were identified by comparison to library entries of purified standards or recurrent unknown entities. Metabolon maintains a library based on authenticated standards that contains the retention time/index (RI), mass to charge ratio (*m/z)*, and chromatographic data (including MS/MS spectral data) on all molecules present in the library. Furthermore, biochemical identifications are based on three criteria: retention index within a narrow RI window of the proposed identification, accurate mass match to the library +/- 10 ppm, and the MS/MS forward and reverse scores between the experimental data and authentic standards. The MS/MS scores are based on a comparison of the ions present in the experimental spectrum to the ions present in the library spectrum. While there may be similarities between these molecules based on one of these factors, the use of all three data points can be utilized to distinguish and differentiate biochemicals. More than 3300 commercially available purified standard compounds have been acquired and registered into LIMS for analysis on all platforms for determination of their analytical characteristics. Additional mass spectral entries have been created for structurally unnamed biochemicals, which have been identified by virtue of their recurrent nature (both chromatographic and mass spectral). These compounds have the potential to be identified by future acquisition of a matching purified standard or by classical structural analysis.

Curation: A variety of curation procedures were carried out to ensure that a high quality data set was made available for statistical analysis and data interpretation. The QC and curation processes were designed to ensure accurate and consistent identification of true chemical entities, and to remove those representing system artifacts, mis-assignments, and background noise. Metabolon data analysts use proprietary visualization and interpretation software to confirm the consistency of peak identification among the various samples. Library matches for each compound were checked for each sample and corrected if necessary.

Metabolite Quantification and Data Normalization: Peaks were quantified using area-under-the-curve. For studies spanning multiple days, a data normalization step was performed to correct variation resulting from instrument inter-day tuning differences. Essentially, each compound was corrected in run-day blocks by registering the medians to equal one (1.00) and normalizing each data point proportionately i.e., "block correction". For studies that did not require more than one day of analysis, no normalization is necessary, other than for purposes of data visualization. In certain instances, biochemical data may have been normalized to an additional factor (e.g., cell counts, total protein as determined by Bradford assay, osmolality, etc.) to account for differences in metabolite levels due to differences in the amount of material present in each sample.

Complex Lipids Platform: Plasma

Lipids were extracted from samples in methanol:dichloromethane in the presence of internal standards. The extracts were concentrated under nitrogen and reconstituted in 0.25mL of 10mM ammonium acetate dichloromethane:methanol (50:50). The extracts were transferred to inserts and placed in vials for infusion-MS analysis, performed on a Shimazdu LC with nano PEEK tubing and the Sciex SelexIon-5500 QTRAP. The samples were analyzed via both positive and negative mode electrospray. The 5500 QTRAP scan was performed in MRM mode with the total of more than 1,100 MRMs. Individual lipid species were quantified by taking the peak area ratios of target compounds and their assigned internal standards, then multiplying by the concentration of internal standard added to the sample. Lipid class concentrations were calculated from the sum of all molecular species within a class, and fatty acid compositions were determined by calculating the proportion of each class comprised by individual fatty acids.

Data Quality: Instrument and Process Variability

Urine Samples

Overall process variability was determined by calculating the median relative standard deviation (RSD) for all endogenous metabolites (i.e., non-instrument standards) present in the CMTRX technical replicates. Values for instrument (6%) and process (7%) variability met Metabolon's acceptance criteria.

Plasma Samples

Overall process variability was determined by calculating the median RSD for all endogenous metabolites (i.e., non-instrument standards) present in the MTRX7 technical replicates. Values for instrument (6%) and process (11%) variability met Metabolon's acceptance criteria.

Supplemental Table 2. Regression estimates of the associations of UPF intake with urine metabolites in adolescents

Metabolite	Model 1, β (95% CI)	Model 2, β (95% CI)	Model 3, β (95% CI)[1]
X - 17679	0.026 (0.021, 0.032)	0.026 (0.020, 0.032)	0.027 (0.021, 0.033)
X - 19497	0.013 (0.007, 0.019)	0.014 (0.008, 0.020)	0.016 (0.010, 0.022)
glucuronide of C10H18O2 (1)*	0.014 (0.007, 0.020)	0.014 (0.007, 0.020)	0.015 (0.008, 0.021)
glucuronide of C10H14O2 (2)*	0.013 (0.007, 0.019)	0.013 (0.007, 0.020)	0.015 (0.008, 0.021)
X - 12818	-0.013 (-0.019, -0.007)	-0.013 (-0.019, -0.007)	-0.014 (-0.021, -0.008)
X - 11478	0.012 (0.006, 0.018)	0.013 (0.006, 0.019)	0.014 (0.007, 0.020)
3-methyladipate	-0.016 (-0.022, -0.010)	-0.013 (-0.019, -0.007)	-0.013 (-0.019, -0.007)
glucuronide of C10H18O2 (7)*	0.012 (0.006, 0.018)	0.012 (0.006, 0.019)	0.014 (0.007, 0.021)
indoxyl glucuronide	0.005 (-0.001, 0.011)	0.010 (0.004, 0.016)	0.013 (0.007, 0.019)
glucuronide of C10H18O2 (8)*	0.013 (0.007, 0.019)	0.012 (0.006, 0.019)	0.014 (0.007, 0.020)
N,N-dimethylalanine	0.010 (0.004, 0.016)	0.012 (0.006, 0.018)	0.013 (0.007, 0.019)
X - 13844	-0.015 (-0.021, -0.009)	-0.013 (-0.019, -0.007)	-0.013 (-0.019, -0.006)
X - 21807	-0.014 (-0.020, -0.008)	-0.013 (-0.019, -0.006)	-0.013 (-0.019, -0.006)
caffeic acid sulfate	-0.014 (-0.020, -0.008)	-0.013 (-0.019, -0.007)	-0.013 (-0.019, -0.006)
hydroxy-N6,N6,N6-trimethyllysine*	0.012 (0.006, 0.018)	0.012 (0.006, 0.018)	0.012 (0.006, 0.018)
4-hydroxycinnamate sulfate	-0.013 (-0.019, -0.007)	-0.012 (-0.019, -0.006)	-0.013 (-0.019, -0.006)
2S,3R-dihydroxybutyrate	-0.014 (-0.019, -0.008)	-0.013 (-0.018, -0.007)	-0.011 (-0.017, -0.005)
1-methylhistamine	0.011 (0.005, 0.016)	0.011 (0.005, 0.017)	0.012 (0.006, 0.018)
glucuronide of C10H18O2 (9)*	0.011 (0.005, 0.017)	0.011 (0.005, 0.017)	0.012 (0.006, 0.019)
heptenedioate (C7:1-DC)*	-0.015 (-0.021, -0.009)	-0.012 (-0.018, -0.006)	-0.012 (-0.018, -0.005)
hydroquinone sulfate	-0.012 (-0.019, -0.006)	-0.011 (-0.017, -0.005)	-0.012 (-0.019, -0.006)
X - 25442	0.011 (0.005, 0.017)	0.013 (0.007, 0.019)	0.012 (0.006, 0.019)
4-hydroxymandelate	-0.011 (-0.017, -0.005)	-0.013 (-0.019, -0.007)	-0.012 (-0.018, -0.005)
4-methoxyphenol sulfate	-0.012 (-0.018, -0.006)	-0.012 (-0.018, -0.006)	-0.012 (-0.018, -0.005)
allantoin	-0.010 (-0.016, -0.004)	-0.011 (-0.017, -0.005)	-0.012 (-0.018, -0.005)
picolinoylglycine	-0.012 (-0.018, -0.007)	-0.012 (-0.018, -0.006)	-0.011 (-0.017, -0.005)
tiglyl carnitine (C5)	-0.009 (-0.015, -0.003)	-0.012 (-0.018, -0.007)	-0.011 (-0.017, -0.005)
X - 17825	0.009 (0.003, 0.015)	0.011 (0.004, 0.017)	0.011 (0.005, 0.017)
X - 24345	0.010 (0.004, 0.016)	0.012 (0.006, 0.018)	0.012 (0.005, 0.018)

X - 17358	0.011 (0.005, 0.017)	0.010 (0.004, 0.017)	0.011 (0.005, 0.018)
dopamine 3-O-sulfate	-0.012 (-0.018, -0.006)	-0.012 (-0.018, -0.006)	-0.011 (-0.018, -0.005)
X - 23459	-0.011 (-0.017, -0.005)	-0.011 (-0.017, -0.005)	-0.011 (-0.018, -0.005)
1,6-anhydroglucose	0.010 (0.004, 0.016)	0.011 (0.005, 0.017)	0.011 (0.004, 0.018)
2-acetamidophenol sulfate	-0.010 (-0.016, -0.004)	-0.011 (-0.017, -0.004)	-0.011 (-0.017, -0.004)
3,5-dihydroxybenzoic acid	-0.012 (-0.018, -0.006)	-0.012 (-0.018, -0.005)	-0.011 (-0.017, -0.004)
3-indoxyl sulfate	0.003 (-0.003, 0.009)	0.008 (0.002, 0.014)	0.010 (0.004, 0.016)
ferulic acid 4-sulfate	-0.011 (-0.017, -0.005)	-0.011 (-0.018, -0.005)	-0.011 (-0.017, -0.004)
glycerophosphorylcholine (GPC)	0.005 (-0.001, 0.011)	0.009 (0.002, 0.015)	0.011 (0.004, 0.017)
glycolate (hydroxyacetate)	0.012 (0.006, 0.018)	0.010 (0.004, 0.016)	0.010 (0.004, 0.016)
X - 13695	-0.009 (-0.015, -0.003)	-0.010 (-0.017, -0.004)	-0.011 (-0.018, -0.004)
6-bromotryptophan	0.011 (0.005, 0.017)	0.010 (0.004, 0.016)	0.010 (0.004, 0.016)
X - 21258	-0.010 (-0.016, -0.004)	-0.010 (-0.016, -0.004)	-0.011 (-0.017, -0.004)

[1]statistically significant results (FDR q-value <0.05).
Model 1: Unadjusted.
Model 2: Adjusted for age, sex, body mass index, and energy intake
Model 3: Adjustments in Model 2 and physical activity, alcohol and smoking status.
Each model was run independently (i.e., separately not based on statistical significance in previous model), overall significance was based on Model 3.
*Indicates a compound that has not been confirmed based on authentic chemical standard, but Metabolon are confident in its identity. The structural identities of 'X-' followed by a number (e.g., X - 11372) are unknown.
Abbreviations: β, regression estimate; CI, confidence intervals; FDR, false discovery rate according to Benjamini-Hochberg.

Supplemental Table 3. Regression estimates of the associations of UPF intake with urine metabolite patterns in adolescents

Metabolite pattern	Model 1, β (95% CI)	Model 2, β (95% CI)	Model 3, β (95% CI)
MP1	-0.045 (-0.100, 0.011)	-0.000 (-0.054, 0.054)	0.009 (-0.048, 0.066)
MP2	-0.002 (-0.044, 0.041)	-0.006 (-0.049, 0.037)	-0.008 (-0.053, 0.037)
MP3	-0.034 (-0.069, 0.001)	-0.036 (-0.072, -0.001)	-0.040 (-0.078, -0.003)
MP4	0.030 (-0.004, 0.064)	-0.005 (-0.032, 0.022)	-0.001 (-0.029, 0.028)
MP5	-0.039 (-0.072, -0.005)	-0.018 (-0.051, 0.015)	-0.022 (-0.057, 0.013)
MP6	-0.017 (-0.048, 0.014)	-0.002 (-0.033, 0.030)	0.013 (-0.019, 0.046)
**MP7**	**-0.062 (-0.090, -0.034)[1]**	**-0.064 (-0.092, -0.036)[1]**	**-0.063 (-0.092, -0.034)[1]**
MP8	0.033 (0.006, 0.059)	0.021 (-0.006, 0.048)	0.025 (-0.004, 0.053)
**MP9**	**0.046 (0.020, 0.072)[1]**	**0.046 (0.019, 0.073)[1]**	**0.042 (0.014, 0.070)[1]**
**MP10**	**0.022 (-0.003, 0.047)**	**0.036 (0.011, 0.061)[1]**	0.029 (0.003, 0.055)
MP11	-0.021 (-0.046, 0.005)	-0.012 (-0.038, 0.015)	-0.013 (-0.041, 0.014)
**MP12**	**-0.035 (-0.059, -0.011)[1]**	-0.022 (-0.045, 0.001)	-0.018 (-0.042, 0.006)
MP13	0.018 (-0.004, 0.041)	0.019 (-0.004, 0.043)	0.021 (-0.003, 0.045)
MP14	0.001 (-0.020, 0.022)	0.006 (-0.015, 0.027)	0.006 (-0.016, 0.028)
MP15	0.001 (-0.018, 0.021)	-0.006 (-0.026, 0.014)	-0.008 (-0.029, 0.014)
MP16	-0.004 (-0.023, 0.016)	-0.008 (-0.027, 0.012)	-0.006 (-0.026, 0.015)
MP17	-0.003 (-0.023, 0.016)	-0.008 (-0.028, 0.012)	-0.012 (-0.033, 0.009)
**MP18**	**-0.020 (-0.042, 0.002)**	**-0.032 (-0.055, -0.010)[1]**	-0.032 (-0.056, -0.008)
MP19	0.002 (-0.017, 0.020)	-0.002 (-0.020, 0.017)	-0.002 (-0.022, 0.017)
MP20	-0.015 (-0.032, 0.002)	-0.015 (-0.032, 0.003)	-0.015 (-0.034, 0.004)
MP21	0.003 (-0.014, 0.021)	-0.000 (-0.019, 0.018)	-0.002 (-0.021, 0.018)
MP22	-0.002 (-0.018, 0.015)	0.003 (-0.014, 0.020)	0.009 (-0.009, 0.026)
MP23	-0.015 (-0.032, 0.002)	-0.007 (-0.024, 0.010)	-0.005 (-0.022, 0.013)
MP24	-0.012 (-0.030, 0.005)	-0.005 (-0.023, 0.013)	-0.008 (-0.027, 0.011)
MP25	0.011 (-0.005, 0.027)	0.016 (0.000, 0.032)	0.016 (-0.001, 0.033)

[1]statistically significant results (FDR q-value <0.05); main results in the manuscript based on Model 3.
Model 1: Unadjusted.
Model 2: Adjusted for age, sex, body mass index, and energy intake
Model 3: Adjustments in Model 2 and physical activity, alcohol and smoking status.
Abbreviations: β, regression estimate; CI, confidence intervals; FDR, false discovery rate according to Benjamini-Hochberg, MP, metabolite pattern.

Supplemental Table 4: Metabolites with non-zero loadings in urine MP9.

super_pathway	sub_pathway	metabolite	Loadings	Hmdb
Amino Acid	Tyrosine Metabolism	dopamine 3-O-sulfate	-0.111	HMDB06275
Amino Acid	Histidine Metabolism	N-acetyl-1-methylhistidine*	0.103	Unknown
Amino Acid	Tyrosine Metabolism	dopamine 4-sulfate	-0.094	HMDB04148
Amino Acid	Alanine and Aspartate Metabolism	asparagine	-0.089	HMDB00168
Amino Acid	Histidine Metabolism	1-methylhistidine	0.083	HMDB00001
Amino Acid	Glutamate Metabolism	glutamine	-0.083	HMDB00641
Amino Acid	Urea cycle; Arginine and Proline Metabolism	N-methylproline	-0.076	Unknown
Amino Acid	Glycine, Serine and Threonine Metabolism	glycine	-0.074	HMDB00123
Amino Acid	Glycine, Serine and Threonine Metabolism	serine	-0.067	HMDB00187
Amino Acid	Methionine, Cysteine, SAM and Taurine Metabolism	cystathionine	-0.063	HMDB00099
Amino Acid	Tryptophan Metabolism	5-hydroxypicolinic acid	0.063	Unknown
Amino Acid	Glycine, Serine and Threonine Metabolism	N-acetylserine	-0.061	HMDB02931
Amino Acid	Glycine, Serine and Threonine Metabolism	betaine	-0.054	HMDB00043
Amino Acid	Glycine, Serine and Threonine Metabolism	N-acetylthreonine	-0.052	HMDB62557
Amino Acid	Methionine, Cysteine, SAM and Taurine Metabolism	2,3-dihydroxy-5-methylthio-4-pentenoate (DMTPA)*	-0.045	Unknown
Amino Acid	Tyrosine Metabolism	catechol glucuronide	0.043	Unknown
Amino Acid	Tyrosine Metabolism	4-methoxyphenol sulfate	-0.043	Unknown
Amino Acid	Tryptophan Metabolism	5-hydroxyindoleacetate	-0.036	HMDB00763
Amino Acid	Urea cycle; Arginine and Proline Metabolism	citrulline	-0.034	HMDB00904
Amino Acid	Tyrosine Metabolism	3,4-dihydroxyphenylacetate	-0.034	HMDB01336
Amino Acid	Tyrosine Metabolism	tyrosine	-0.033	HMDB00158
Amino Acid	Tryptophan Metabolism	tryptophan	-0.027	HMDB00929
Amino Acid	Urea cycle; Arginine and Proline Metabolism	N-acetylhomocitrulline	0.026	Unknown
Amino Acid	Glutamate Metabolism	N-methylglutamate	-0.026	Unknown
Amino Acid	Glycine, Serine and Threonine Metabolism	dimethylglycine	-0.023	HMDB00092
Amino Acid	Lysine Metabolism	lysine	-0.022	HMDB00182
Amino Acid	Leucine, Isoleucine and Valine Metabolism	N-carbamoylvaline	0.021	Unknown
Amino Acid	Glutamate Metabolism	alpha-ketoglutaramate*	-0.017	Unknown
Amino Acid	Glycine, Serine and Threonine Metabolism	threonine	-0.017	HMDB00167
Amino Acid	Alanine and Aspartate Metabolism	alanine	-0.014	HMDB00161
Amino Acid	Urea cycle; Arginine and Proline Metabolism	N-methylhydroxyproline	-0.012	Unknown
Amino Acid	Lysine Metabolism	N2-acetyllysine	0.012	HMDB00446
Amino Acid	Tyrosine Metabolism	3-methoxytyramine sulfate	-0.012	Unknown
Amino Acid	Tyrosine Metabolism	vanillic alcohol sulfate	0.01	Unknown

Amino Acid	Tyrosine Metabolism	homovanillate sulfate	-0.007	HMDB11719
Amino Acid	Alanine and Aspartate Metabolism	N-carbamoylalanine	0.006	Unknown
Amino Acid	Lysine Metabolism	fructosyllysine	-0.005	Unknown
Amino Acid	Tryptophan Metabolism	N-acetyltryptophan	-0.004	HMDB13713
Amino Acid	Tyrosine Metabolism	3,4-dihydroxyphenylacetate sulfate	-0.003	Unknown
Amino Acid	Lysine Metabolism	6-oxopiperidine-2-carboxylate	0.003	HMDB61705
Carbohydrate	Pentose Metabolism	ribonate (ribonolactone)	0.004	HMDB00867
Cofactors and Vitamins	Pantothenate and CoA Metabolism	pantothenate (Vitamin B5)	-0.047	HMDB00210
Cofactors and Vitamins	Thiamine Metabolism	thiamin (Vitamin B1)	-0.034	HMDB00235
Cofactors and Vitamins	Nicotinate and Nicotinamide Metabolism	nicotinamide N-oxide	0.027	HMDB02730
Cofactors and Vitamins	Nicotinate and Nicotinamide Metabolism	trigonelline (N'-methylnicotinate)	0.005	HMDB00875
Energy	TCA Cycle	citraconate/glutaconate	0.169	Unknown
Energy	TCA Cycle	citrate	-0.089	HMDB00094
Energy	TCA Cycle	fumarate	-0.055	HMDB00134
Lipid	Fatty Acid, Monohydroxy	3-hydroxyhexanoate	-0.106	Unknown
Lipid	Fatty Acid, Dicarboxylate	azelate (nonanedioate; C9)	-0.087	HMDB00784
Lipid	Fatty Acid, Dicarboxylate	pimelate (C7-DC)	-0.071	HMDB00857
Lipid	Fatty Acid, Dicarboxylate	2-hydroxysebacate	-0.059	HMDB00424
Lipid	Fatty Acid Metabolism (Acyl Carnitine, Monounsaturated)	5-dodecenoylcarnitine (C12:1)	0.035	HMDB13326
Lipid	Fatty Acid, Dicarboxylate	sebacate (C10-DC)	-0.024	HMDB00792
Lipid	Fatty Acid, Monohydroxy	3-hydroxysebacate	-0.021	HMDB00350
Lipid	Androgenic Steroids	androsterone sulfate	-0.02	HMDB02759
Lipid	Inositol Metabolism	chiro-inositol	-0.019	HMDB34220
Lipid	Fatty Acid, Dicarboxylate	maleate	0.019	HMDB00176
Lipid	Progestin Steroids	pregnanediol-3-glucuronide	-0.017	HMDB10318
Lipid	Fatty Acid, Dicarboxylate	2-hydroxyadipate	0.012	HMDB00321
Lipid	Progestin Steroids	5alpha-pregnan-diol disulfate	-0.011	Unknown
Lipid	Fatty Acid Metabolism (Acyl Glycine)	2-butenoylglycine	-0.011	Unknown
Lipid	Phospholipid Metabolism	choline	-0.01	HMDB00097
Lipid	Fatty Acid, Dicarboxylate	4-octenedioate	-0.001	HMDB04982
Nucleotide	Purine Metabolism, (Hypo)Xanthine/Inosine containing	allantoin	-0.081	HMDB00462
Nucleotide	Purine Metabolism, (Hypo)Xanthine/Inosine containing	N1-methylinosine	0.035	HMDB02721
Nucleotide	Purine Metabolism, Adenine containing	N6-carbamoylthreonyladenosine	0.033	HMDB41623
Nucleotide	Pyrimidine Metabolism, Orotate containing	orotidine	0.028	HMDB00788
Nucleotide	Pyrimidine Metabolism, Cytidine containing	cytidine	0.011	HMDB00089
PCM	Partially Characterized Molecules	glucuronide of C14H26O4 (1)*	-0.105	Unknown
PCM	Partially Characterized Molecules	glucuronide of C14H26O4 (2)*	-0.075	Unknown
PCM	Partially Characterized Molecules	glucuronide of C12H22O4 (1)*	-0.054	Unknown

PCM	Partially Characterized Molecules	pentose acid*	-0.05	Unknown
PCM	Partially Characterized Molecules	glucuronide of C8H14O2 (6)*	-0.035	Unknown
PCM	Partially Characterized Molecules	glucuronide of C8H18O2 (2)*	0.029	Unknown
PCM	Partially Characterized Molecules	glucuronide of C12H22O4 (2)*	-0.02	Unknown
PCM	Partially Characterized Molecules	glucuronide of C12H22O3 (1)*	-0.016	Unknown
PCM	Partially Characterized Molecules	glucuronide of C8H16O2 (2)*	-0.013	Unknown
PCM	Partially Characterized Molecules	glucuronide of C10H14O2 (2)*	0.01	Unknown
PCM	Partially Characterized Molecules	glucuronide of C10H18O2 (10)*	-0.001	Unknown
Peptide	Dipeptide	cyclo(pro-hydroxypro)*	0.069	Unknown
Peptide	Acetylated Peptides	phenylacetylleucine	0.048	Unknown
Peptide	Gamma-glutamyl Amino Acid	gamma-glutamylphenylalanine	-0.042	HMDB00594
Peptide	Gamma-glutamyl Amino Acid	gamma-glutamylglycine	-0.028	HMDB11667
Peptide	Gamma-glutamyl Amino Acid	gamma-glutamylleucine	-0.022	HMDB11171
Peptide	Dipeptide	valylleucine	0.02	HMDB29131
Peptide	Dipeptide	cyclo(pro-tyr)	0.011	Unknown
Unknown	Unknown	X - 12738	0.25	Unknown
Unknown	Unknown	X - 23655	0.244	Unknown
Unknown	Unknown	X - 24811	0.199	Unknown
Unknown	Unknown	X - 24499	0.155	Unknown
Unknown	Unknown	X - 12722	-0.146	Unknown
Unknown	Unknown	X - 17010	-0.146	Unknown
Unknown	Unknown	X - 24344	0.129	Unknown
Unknown	Unknown	X - 17688	-0.123	Unknown
Unknown	Unknown	X - 13866	-0.109	Unknown
Unknown	Unknown	X - 12818	-0.109	Unknown
Unknown	Unknown	X - 12306	-0.081	Unknown
Unknown	Unknown	X - 23581	-0.077	Unknown
Unknown	Unknown	X - 24343	0.076	Unknown
Unknown	Unknown	X - 15666	-0.066	Unknown
Unknown	Unknown	X - 17438	-0.064	Unknown
Unknown	Unknown	X - 17398	0.063	Unknown
Unknown	Unknown	X - 17343	-0.058	Unknown
Unknown	Unknown	X - 24349	0.057	Unknown
Unknown	Unknown	X - 11640	-0.057	Unknown
Unknown	Unknown	X - 12101	-0.055	Unknown
Unknown	Unknown	X - 17300	0.054	Unknown
Unknown	Unknown	X - 13728	0.054	Unknown
Unknown	Unknown	X - 24736	-0.041	Unknown
Unknown	Unknown	X - 15492	0.039	Unknown
Unknown	Unknown	X - 12906	-0.039	Unknown
Unknown	Unknown	X - 17685	0.038	Unknown
Unknown	Unknown	X - 21807	-0.037	Unknown
Unknown	Unknown	X - 12407	-0.032	Unknown
Unknown	Unknown	X - 18410	0.032	Unknown
Unknown	Unknown	X - 24794	0.031	Unknown
Unknown	Unknown	X - 23459	-0.027	Unknown
Unknown	Unknown	X - 12704	-0.026	Unknown
Unknown	Unknown	X - 22143	0.026	Unknown

Unknown	Unknown	X - 21815	-0.025	Unknown
Unknown	Unknown	X - 17676	0.024	Unknown
Unknown	Unknown	X - 23517	-0.024	Unknown
Unknown	Unknown	X - 12267	-0.024	Unknown
Unknown	Unknown	X - 24457	0.019	Unknown
Unknown	Unknown	X - 24498	0.017	Unknown
Unknown	Unknown	X - 24341	-0.017	Unknown
Unknown	Unknown	X - 23314	-0.016	Unknown
Unknown	Unknown	X - 17704	-0.016	Unknown
Unknown	Unknown	X - 23161	0.014	Unknown
Unknown	Unknown	X - 18888	-0.013	Unknown
Unknown	Unknown	X - 24813	-0.013	Unknown
Unknown	Unknown	X - 13874	-0.011	Unknown
Unknown	Unknown	X - 24542	0.011	Unknown
Unknown	Unknown	X - 24330	-0.01	Unknown
Unknown	Unknown	X - 12007	-0.01	Unknown
Unknown	Unknown	X - 12410	-0.009	Unknown
Unknown	Unknown	X - 17765	-0.009	Unknown
Unknown	Unknown	X - 24801	0.009	Unknown
Unknown	Unknown	X - 17673	0.008	Unknown
Unknown	Unknown	X - 22757	-0.008	Unknown
Unknown	Unknown	X - 12847	0.008	Unknown
Unknown	Unknown	X - 24249	0.007	Unknown
Unknown	Unknown	X - 21821	-0.006	Unknown
Unknown	Unknown	X - 21792	-0.006	Unknown
Unknown	Unknown	X - 24812	0.005	Unknown
Unknown	Unknown	X - 18059	-0.005	Unknown
Unknown	Unknown	X - 23518	0.004	Unknown
Unknown	Unknown	X - 17351	-0.003	Unknown
Unknown	Unknown	X - 17328	0.002	Unknown
Unknown	Unknown	X - 24462	-0.001	Unknown
Xenobiotics	Xanthine Metabolism	1-methylxanthine	0.272	HMDB10738
Xenobiotics	Chemical	3-hydroxypyridine glucuronide	0.252	Unknown
Xenobiotics	Xanthine Metabolism	1,3,7-trimethylurate	0.229	HMDB02123
Xenobiotics	Xanthine Metabolism	1-methylurate	0.215	HMDB03099
Xenobiotics	Xanthine Metabolism	1,7-dimethylurate	0.214	HMDB11103
Xenobiotics	Chemical	3-hydroxypyridine sulfate	0.206	Unknown
Xenobiotics	Xanthine Metabolism	5-acetylamino-6-amino-3-methyluracil	0.19	HMDB04400
Xenobiotics	Food Component/Plant	2-furoylcarnitine	0.182	Unknown
Xenobiotics	Food Component/Plant	3-ethylcatechol sulfate (1)	0.18	Unknown
Xenobiotics	Benzoate Metabolism	3-methyl catechol sulfate (1)	0.179	Unknown
Xenobiotics	Chemical	3-acetylphenol sulfate	0.167	Unknown
Xenobiotics	Xanthine Metabolism	caffeine	0.163	HMDB01847
Xenobiotics	Benzoate Metabolism	3-methyl catechol sulfate (2)	0.147	Unknown
Xenobiotics	Chemical	3-hydroxy-2-methylpyridine sulfate	0.139	Unknown
Xenobiotics	Benzoate Metabolism	4-ethylcatechol sulfate	0.131	Unknown

Xenobiotics	Chemical	5-hydroxy-2-methylpyridine sulfate	0.128	Unknown
Xenobiotics	Benzoate Metabolism	o-cresol sulfate	0.119	Unknown
Xenobiotics	Food Component/Plant	N-(2-furoyl)glycine	0.116	HMDB00439
Xenobiotics	Benzoate Metabolism	2-ethylphenylsulfate	0.085	Unknown
Xenobiotics	Food Component/Plant	3,5-dihydroxybenzoic acid	-0.08	HMDB13677
Xenobiotics	Food Component/Plant	(2,4 or 2,5)-dimethylphenol sulfate	0.076	Unknown
Xenobiotics	Xanthine Metabolism	7-methylxanthine	0.076	HMDB01991
Xenobiotics	Food Component/Plant	stachydrine	-0.071	HMDB04827
Xenobiotics	Food Component/Plant	pyrraline	-0.071	HMDB33143
Xenobiotics	Chemical	2-aminophenol sulfate	-0.069	HMDB61116
Xenobiotics	Xanthine Metabolism	3-methylxanthine	0.068	HMDB01886
Xenobiotics	Xanthine Metabolism	7-methylurate	0.066	HMDB11107
Xenobiotics	Food Component/Plant	ferulic acid 4-sulfate	-0.06	HMDB29200
Xenobiotics	Drug - Topical Agents	2,6-dihydroxybenzoic acid	-0.058	HMDB13676
Xenobiotics	Xanthine Metabolism	3,7-dimethylurate	0.058	HMDB01982
Xenobiotics	Food Component/Plant	sulfate of piperine metabolite C16H19NO3 (3)*	0.058	Unknown
Xenobiotics	Benzoate Metabolism	guaiacol sulfate	0.058	HMDB60013
Xenobiotics	Drug - Analgesics, Anesthetics	2-acetamidophenol sulfate	-0.058	Unknown
Xenobiotics	Xanthine Metabolism	3-methylurate*	0.05	Unknown
Xenobiotics	Food Component/Plant	glucuronide of piperine metabolite C17H21NO3 (6)*	0.04	Unknown
Xenobiotics	Food Component/Plant	sulfate of piperine metabolite C16H19NO3 (1)*	0.04	Unknown
Xenobiotics	Food Component/Plant	vanillate	0.033	HMDB00484
Xenobiotics	Food Component/Plant	3-hydroxystachydrine*	-0.032	Unknown
Xenobiotics	Food Component/Plant	abscisate	-0.029	HMDB35140
Xenobiotics	Food Component/Plant	N-acetylpyrraline	-0.029	Unknown
Xenobiotics	Food Component/Plant	glucuronide of piperine metabolite C17H21NO3 (5)*	0.027	Unknown
Xenobiotics	Xanthine Metabolism	theobromine	0.022	HMDB02825
Xenobiotics	Food Component/Plant	glucuronide of piperine metabolite C17H21NO3 (3)*	0.02	Unknown
Xenobiotics	Food Component/Plant	3-hydroxycinnamate	-0.018	HMDB01713
Xenobiotics	Food Component/Plant	sulfate of piperine metabolite C18H21NO3 (2)*	0.015	Unknown
Xenobiotics	Benzoate Metabolism	2-hydroxyhippurate (salicylurate)	-0.014	HMDB00840
Xenobiotics	Benzoate Metabolism	3-(3-hydroxyphenyl)propionate	-0.014	HMDB00375
Xenobiotics	Food Component/Plant	glucuronide of piperine metabolite C17H21NO3 (2)*	0.012	Unknown
Xenobiotics	Food Component/Plant	4-acetylcatechol sulfate (1)	0.012	Unknown
Xenobiotics	Food Component/Plant	sulfate of piperine metabolite C16H19NO4 (4)*	0.012	Unknown
Xenobiotics	Benzoate Metabolism	2,4,6-trihydroxybenzoate	-0.009	HMDB29649
Xenobiotics	Food Component/Plant	naringenin 7-glucuronide	-0.009	Unknown
Xenobiotics	Food Component/Plant	methyl indole-3-acetate	-0.008	HMDB29738
Xenobiotics	Benzoate Metabolism	4-hydroxymandelate	-0.008	HMDB00822

Xenobiotics	Food Component/Plant	sulfate of piperine metabolite C18H21NO3 (1)*	0.008	Unknown
Xenobiotics	Food Component/Plant	sulfate of piperine metabolite C16H19NO4 (1)*	0.008	Unknown
Xenobiotics	Food Component/Plant	furaneol sulfate	-0.007	Unknown
Xenobiotics	Chemical	(2-butoxyethoxy)acetic acid	-0.007	Unknown
Xenobiotics	Drug - Neurological	3-hydroxyisonicotinic acid	0.005	Unknown
Xenobiotics	Chemical	glycolate (hydroxyacetate)	0.004	HMDB00115
Xenobiotics	Benzoate Metabolism	4-hydroxyhippurate	-0.003	HMDB13678
Xenobiotics	Food Component/Plant	4-acetylcatechol sulfate (2)	0.002	Unknown
Xenobiotics	Food Component/Plant	2,3-dihydroxypyridine	0.001	Unknown

Supplemental Table 5: Metabolites with non-zero loadings in urine MP7.

super_pathway	sub_pathway	metabolite	V7	Hmdb
Amino Acid	Alanine and Aspartate Metabolism	alanine	-0.185	HMDB00161
Amino Acid	Alanine and Aspartate Metabolism	asparagine	-0.124	HMDB00168
Amino Acid	Alanine and Aspartate Metabolism	N-acetylasparagine	-0.049	HMDB06028
Amino Acid	Creatine Metabolism	creatinine	0.126	HMDB00562
Amino Acid	Glutamate Metabolism	4-hydroxyglutamate	-0.075	HMDB0002273
Amino Acid	Glutamate Metabolism	pyroglutamine*	0.045	HMDB0062558
Amino Acid	Glutamate Metabolism	glutamine	-0.14	HMDB00641
Amino Acid	Glutamate Metabolism	N-acetylglutamate	-0.025	HMDB01138
Amino Acid	Glutathione Metabolism	2-aminobutyrate	-0.126	HMDB00650
Amino Acid	Glycine, Serine and Threonine Metabolism	glycine	-0.088	HMDB00123
Amino Acid	Glycine, Serine and Threonine Metabolism	threonine	-0.236	HMDB00167
Amino Acid	Glycine, Serine and Threonine Metabolism	serine	-0.084	HMDB00187
Amino Acid	Glycine, Serine and Threonine Metabolism	sarcosine	-0.002	HMDB00271
Amino Acid	Glycine, Serine and Threonine Metabolism	N-acetylglycine	-0.108	HMDB00532
Amino Acid	Glycine, Serine and Threonine Metabolism	N-acetylserine	-0.005	HMDB02931
Amino Acid	Glycine, Serine and Threonine Metabolism	2-methylserine	-0.106	Uknown
Amino Acid	Guanidino and Acetamido Metabolism	guanidinosuccinate	0.073	HMDB03157
Amino Acid	Histidine Metabolism	histidine	-0.022	HMDB00177
Amino Acid	Histidine Metabolism	histamine	-0.043	HMDB00870
Amino Acid	Histidine Metabolism	1-methylhistamine	-0.014	HMDB00898
Amino Acid	Histidine Metabolism	hydantoin-5-propionate	0.119	HMDB01212
Amino Acid	Histidine Metabolism	4-imidazoleacetate	0.016	HMDB02024
Amino Acid	Histidine Metabolism	imidazole propionate	-0.004	HMDB02271
Amino Acid	Histidine Metabolism	1-methyl-4-imidazoleacetate	0.008	HMDB02820
Amino Acid	Histidine Metabolism	N-acetylcarnosine	0.058	HMDB12881
Amino Acid	Histidine Metabolism	N-acetylhistidine	-0.036	HMDB32055
Amino Acid	Leucine, Isoleucine and Valine Metabolism	2-hydroxy-3-methylvalerate	-0.005	HMDB00317
Amino Acid	Leucine, Isoleucine and Valine Metabolism	3-hydroxyisobutyrate	-0.03	HMDB00336
Amino Acid	Leucine, Isoleucine and Valine Metabolism	2-methylbutyrylglycine (C5)	-0.028	HMDB00339
Amino Acid	Leucine, Isoleucine and Valine Metabolism	leucine	-0.113	HMDB00687
Amino Acid	Leucine, Isoleucine and Valine Metabolism	beta-hydroxyisovalerate	-0.031	HMDB00754
Amino Acid	Leucine, Isoleucine and Valine Metabolism	valine	-0.183	HMDB00883
Amino Acid	Leucine, Isoleucine and Valine Metabolism	tiglyl carnitine (C5)	0.041	HMDB02366
Amino Acid	Leucine, Isoleucine and Valine Metabolism	N-acetylleucine	-0.105	HMDB11756
Amino Acid	Leucine, Isoleucine and Valine Metabolism	N-acetylvaline	-0.07	HMDB11757

Amino Acid	Leucine, Isoleucine and Valine Metabolism	1-carboxyethylisoleucine	-0.113	Uknown
Amino Acid	Leucine, Isoleucine and Valine Metabolism	1-carboxyethylleucine	-0.1	Uknown
Amino Acid	Leucine, Isoleucine and Valine Metabolism	2,3-dimethylsuccinate	0.005	Uknown
Amino Acid	Lysine Metabolism	lysine	-0.02	HMDB00182
Amino Acid	Lysine Metabolism	glutaroylcarnitine (C5)	0.016	HMDB13130
Amino Acid	Lysine Metabolism	hydroxy-N6,N6,N6-trimethyllysine*	-0.013	Uknown
Amino Acid	Lysine Metabolism	5-(galactosylhydroxy)-L-lysine	-0.004	Uknown
Amino Acid	Methionine, Cysteine, SAM and Taurine Metabolism	taurine	0.037	HMDB00251
Amino Acid	Methionine, Cysteine, SAM and Taurine Metabolism	methionine sulfone	-0.026	HMDB0062174
Amino Acid	Methionine, Cysteine, SAM and Taurine Metabolism	S-adenosylhomocysteine (SAH)	-0.009	HMDB00939
Amino Acid	Methionine, Cysteine, SAM and Taurine Metabolism	S-adenosylmethionine (SAM)	-0.06	HMDB01185
Amino Acid	Methionine, Cysteine, SAM and Taurine Metabolism	methionine sulfoxide	-0.065	HMDB02005
Amino Acid	Methionine, Cysteine, SAM and Taurine Metabolism	N-acetylmethionine sulfoxide	-0.033	HMDB0240343
Amino Acid	Phenylalanine Metabolism	phenylalanine	-0.116	HMDB00159
Amino Acid	Phenylalanine Metabolism	N-acetylphenylalanine	-0.022	HMDB00512
Amino Acid	Polyamine Metabolism	spermidine	-0.014	HMDB01257
Amino Acid	Polyamine Metabolism	N-acetylputrescine	-0.011	HMDB02064
Amino Acid	Polyamine Metabolism	N1,N12-diacetylspermine	-0.076	HMDB02172
Amino Acid	Tryptophan Metabolism	indoleacetate	-0.03	HMDB00197
Amino Acid	Tryptophan Metabolism	tryptophan	-0.108	HMDB00929
Amino Acid	Tryptophan Metabolism	indoleacetylglutamine	-0.037	HMDB13240
Amino Acid	Tryptophan Metabolism	N-acetyltryptophan	-0.078	HMDB13713
Amino Acid	Tryptophan Metabolism	6-bromotryptophan	-0.189	Uknown
Amino Acid	Tryptophan Metabolism	5-hydroxypicolinic acid	-0.079	Uknown
Amino Acid	Tryptophan Metabolism	C-glycosyltryptophan	-0.076	Uknown
Amino Acid	Tryptophan Metabolism	N-acetylkynurenine (2)	-0.035	Uknown
Amino Acid	Tryptophan Metabolism	7-hydroxyindole sulfate	0.007	Uknown
Amino Acid	Tyrosine Metabolism	homovanillate (HVA)	-0.098	HMDB00118
Amino Acid	Tyrosine Metabolism	vanillic alcohol sulfate	-0.002	Uknown
Amino Acid	Urea cycle; Arginine and Proline Metabolism	proline	-0.055	HMDB00162
Amino Acid	Urea cycle; Arginine and Proline Metabolism	homoarginine	-0.023	HMDB00670
Amino Acid	Urea cycle; Arginine and Proline Metabolism	hydroxyproline	-0.036	HMDB00725
Amino Acid	Urea cycle; Arginine and Proline Metabolism	citrulline	-0.069	HMDB00904
Amino Acid	Urea cycle; Arginine and Proline Metabolism	dimethylarginine	-0.077	HMDB01539
Amino Acid	Urea cycle; Arginine and Proline Metabolism	symmetric dimethylarginine (SDMA)	-0.014	HMDB03334
Carbohydrate	Aminosugar Metabolism	glucuronate	0.097	HMDB00127
Carbohydrate	Aminosugar Metabolism	N-acetylglucosaminylasparagine	-0.005	HMDB00489
Carbohydrate	Aminosugar Metabolism	erythronate*	0.062	HMDB00613

Carbohydrate	Pentose Metabolism	ribonate (ribonolactone)	-0.04	HMDB00867
Cofactors and Vitamins	Ascorbate and Aldarate Metabolism	threonate	0.003	HMDB00943
Cofactors and Vitamins	Pterin Metabolism	pterin	-0.026	HMDB00802
Cofactors and Vitamins	Tocopherol Metabolism	gamma-CEHC	-0.106	HMDB01931
Cofactors and Vitamins	Tocopherol Metabolism	gamma-CEHC glucuronide*	-0.129	Uknown
Cofactors and Vitamins	Tocopherol Metabolism	alpha-CEHC glucuronide*	-0.071	Uknown
Cofactors and Vitamins	Tocopherol Metabolism	delta-CEHC sulfate	-0.068	Uknown
Cofactors and Vitamins	Tocopherol Metabolism	gamma-CEHC taurine*	-0.043	Uknown
Energy	Oxidative Phosphorylation	phosphate	0.003	HMDB01429
Energy	TCA Cycle	cis-aconitate	0.032	HMDB00072
Energy	TCA Cycle	isocitrate	0.074	HMDB00193
Energy	TCA Cycle	2-methylcitrate	0.104	HMDB00379
Lipid	Androgenic Steroids	5alpha-androstan-3beta,17beta-diol disulfate	0.056	HMDB00493
Lipid	Androgenic Steroids	epiandrosterone sulfate	0.065	HMDB0062657
Lipid	Androgenic Steroids	dehydroepiandrosterone sulfate (DHEA-S)	0.068	HMDB01032
Lipid	Androgenic Steroids	androsterone glucuronide	0.019	HMDB02829
Lipid	Androgenic Steroids	androstenediol (3beta,17beta) disulfate (1)	0.094	HMDB03818
Lipid	Androgenic Steroids	androstenediol (3beta,17beta) disulfate (2)	0.082	HMDB03818
Lipid	Androgenic Steroids	androstenediol (3beta,17beta) monosulfate (1)	0.051	HMDB03818
Lipid	Androgenic Steroids	etiocholanolone glucuronide	0.028	HMDB04484
Lipid	Androgenic Steroids	5alpha-androstan-3beta,17alpha-diol disulfate	0.012	Uknown
Lipid	Androgenic Steroids	epiandrosterone glucuronide	0.009	Uknown
Lipid	Carnitine Metabolism	carnitine	0.01	HMDB00062
Lipid	Carnitine Metabolism	deoxycarnitine	0.033	HMDB01161
Lipid	Corticosteroids	cortisone	0.08	HMDB02802
Lipid	Corticosteroids	cortolone	-0.04	HMDB03128
Lipid	Endocannabinoid	hexanoyltaurine	-0.04	Uknown
Lipid	Fatty Acid Metabolism (Acyl Carnitine, Hydroxy)	(S)-3-hydroxybutyrylcarnitine	0.168	HMDB13127
Lipid	Fatty Acid Metabolism (Acyl Carnitine, Hydroxy)	3-hydroxyhexanoylcarnitine (1)	0.103	Uknown
Lipid	Fatty Acid Metabolism (Acyl Carnitine, Monounsaturated)	butenoylcarnitine (C4:1)	0.04	Uknown
Lipid	Fatty Acid Metabolism (Acyl Carnitine, Short Chain)	acetylcarnitine (C2)	0.02	HMDB00201
Lipid	Fatty Acid Metabolism (Acyl Glycine)	N-octanoylglycine	-0.001	HMDB00832
Lipid	Fatty Acid Metabolism (Acyl Glycine)	3-hydroxybutyroylglycine	0.158	Uknown
Lipid	Fatty Acid Metabolism (Acyl Glycine)	trans-2-hexenoylglycine	0.03	Uknown
Lipid	Fatty Acid Synthesis	malonylcarnitine	0.111	HMDB02095

Lipid	Fatty Acid Synthesis	2-methylmalonylcarnitine (C4-DC)	0.03	HMDB13133
Lipid	Fatty Acid, Dicarboxylate	2-hydroxyadipate	0.046	HMDB00321
Lipid	Fatty Acid, Dicarboxylate	3-hydroxyadipate*	0.059	HMDB00345
Lipid	Fatty Acid, Dicarboxylate	pimelate (C7-DC)	-0.014	HMDB00857
Lipid	Fatty Acid, Dicarboxylate	suberate (C8-DC)	-0.045	HMDB00893
Lipid	Fatty Acid, Dicarboxylate	heptenedioate (C7:1-DC)*	0.2	Uknown
Lipid	Fatty Acid, Dihydroxy	2S,3R-dihydroxybutyrate	0.254	HMDB02453
Lipid	Fatty Acid, Monohydroxy	3-hydroxysuberate	0.061	HMDB00325
Lipid	Fatty Acid, Monohydroxy	3-hydroxypropanoate	-0.063	HMDB00700
Lipid	Phosphatidylcholine (PC)	1-palmitoyl-2-oleoyl-GPC (16:0/18:1)	-0.098	HMDB0007972
Lipid	Phosphatidylcholine (PC)	1-palmitoyl-2-linoleoyl-GPC (16:0/18:2)	-0.075	HMDB0007973
Lipid	Phospholipid Metabolism	glycerophosphorylcholine (GPC)	-0.034	HMDB00086
Lipid	Phospholipid Metabolism	glycerophosphoinositol*	-0.086	HMDB0011649
Lipid	Phospholipid Metabolism	trimethylamine N-oxide	0.024	HMDB00925
Lipid	Plasmalogen	1-(1-enyl-stearoyl)-2-arachidonoyl-GPE (P-18:0/20:4)*	-0.194	HMDB0005779
Lipid	Plasmalogen	1-(1-enyl-palmitoyl)-2-arachidonoyl-GPE (P-16:0/20:4)*	-0.127	HMDB11352
Lipid	Pregnenolone Steroids	pregnenetriol sulfate*	0.018	Uknown
Lipid	Pregnenolone Steroids	pregnen-diol disulfate*	0.011	Uknown
Lipid	Pregnenolone Steroids	21-hydroxypregnenolone disulfate	0.008	Uknown
Lipid	Primary Bile Acid Metabolism	glycocholate	-0.087	HMDB00138
Lipid	Primary Bile Acid Metabolism	glycochenodeoxycholate glucuronide (1)	-0.115	Uknown
Lipid	Primary Bile Acid Metabolism	glycocholate glucuronide (1)	-0.038	Uknown
Lipid	Primary Bile Acid Metabolism	glyco-beta-muricholate	-0.027	Uknown
Lipid	Secondary Bile Acid Metabolism	ursocholate	-0.042	HMDB0000917
Lipid	Secondary Bile Acid Metabolism	12-dehydrocholate	-0.035	HMDB00400
Lipid	Secondary Bile Acid Metabolism	glycodeoxycholate 3-sulfate	-0.036	Uknown
Lipid	Secondary Bile Acid Metabolism	taurodeoxycholic acid 3-sulfate	-0.002	Uknown
Lipid	Sphingomyelins	palmitoyl sphingomyelin (d18:1/16:0)	-0.081	Uknown
Nucleotide	Purine Metabolism, (Hypo)Xanthine/Inosine containing	urate	0.079	HMDB00289
Nucleotide	Purine Metabolism, (Hypo)Xanthine/Inosine containing	xanthine	0.065	HMDB00292
Nucleotide	Purine Metabolism, Adenine containing	adenosine 3',5'-cyclic monophosphate (cAMP)	-0.021	HMDB00058
Nucleotide	Purine Metabolism, Adenine containing	N6-succinyladenosine	-0.027	HMDB00912
Nucleotide	Purine Metabolism, Guanine containing	guanosine-3',5'-cyclic monophosphate (cGMP)	-0.108	HMDB01314
Nucleotide	Purine Metabolism, Guanine containing	1-methylguanosine	0.091	HMDB01563
Nucleotide	Pyrimidine Metabolism, Cytidine containing	cytidine	-0.008	HMDB00089
Nucleotide	Pyrimidine Metabolism, Cytidine containing	cytosine	0.028	HMDB00630

Nucleotide	Pyrimidine Metabolism, Thymine containing	5,6-dihydrothymine	-0.024	HMDB00079
Nucleotide	Pyrimidine Metabolism, Uracil containing	5,6-dihydrouracil	0.028	HMDB00076
Nucleotide	Pyrimidine Metabolism, Uracil containing	N3-methyluridine	-0.013	HMDB04813
Nucleotide	Pyrimidine Metabolism, Uracil containing	3-(3-amino-3-carboxypropyl)uridine*	-0.018	Uknown
PCM	Partially Characterized Molecules	N-acetylglucosamine conjugate of C24H40O4 bile acid	-0.058	Uknown
PCM	Partially Characterized Molecules	glycine conjugate of C9H16O2*	-0.037	Uknown
PCM	Partially Characterized Molecules	N-acetylglucosamine conjugate of C24H38O4 bile acid	-0.028	Uknown
PCM	Partially Characterized Molecules	glucuronide of C8H14O2 (6)*	0.024	Uknown
PCM	Partially Characterized Molecules	glucuronide of C10H18O2 (3)*	0.013	Uknown
PCM	Partially Characterized Molecules	glycine conjugate of C10H12O2*	0.012	Uknown
PCM	Partially Characterized Molecules	glucuronide of C12H22O4 (1)*	-0.003	Uknown
Peptide	Acetylated Peptides	4-hydroxyphenylacetylglycine	0.005	HMDB0000735
Peptide	Acetylated Peptides	phenylacetylglutamate	-0.031	HMDB59772
Peptide	Acetylated Peptides	phenylacetylalanine	-0.056	Uknown
Peptide	Acetylated Peptides	phenylacetylisoleucine	-0.051	Uknown
Peptide	Acetylated Peptides	phenylacetylphenylalanine	-0.026	Uknown
Peptide	Dipeptide	phenylalanylhydroxyproline*	-0.037	HMDB11176
Peptide	Dipeptide	valylleucine	-0.105	HMDB29131
Peptide	Dipeptide	cyclo(pro-tyr)	-0.059	Uknown
Peptide	Dipeptide Derivative	isoleucylhydroxyproline*	-0.043	HMDB28908
Peptide	Dipeptide Derivative	leucylhydroxyproline*	-0.017	HMDB28930
Peptide	Gamma-glutamyl Amino Acid	gamma-glutamylglutamine	-0.185	HMDB11738
Uknown	Uknown	X - 21310	0.175	Uknown
Uknown	Uknown	X - 13729	0.155	Uknown
Uknown	Uknown	X - 12823	-0.139	Uknown
Uknown	Uknown	X - 23166	-0.128	Uknown
Uknown	Uknown	X - 16654	-0.122	Uknown
Uknown	Uknown	X - 22519	0.119	Uknown
Uknown	Uknown	X - 12206	0.116	Uknown
Uknown	Uknown	X - 17370	0.115	Uknown
Uknown	Uknown	X - 18410	-0.111	Uknown
Uknown	Uknown	X - 15469	0.104	Uknown
Uknown	Uknown	X - 24468	0.094	Uknown
Uknown	Uknown	X - 12379	-0.093	Uknown
Uknown	Uknown	X - 17327	0.09	Uknown
Uknown	Uknown	X - 24456	-0.089	Uknown
Uknown	Uknown	X - 24527	0.078	Uknown
Uknown	Uknown	X - 16580	0.076	Uknown
Uknown	Uknown	X - 12101	0.074	Uknown
Uknown	Uknown	X - 12472	0.073	Uknown
Uknown	Uknown	X - 13728	-0.07	Uknown
Uknown	Uknown	X - 23653	0.067	Uknown
Uknown	Uknown	X - 24406	0.063	Uknown

Uknown	Uknown	X - 11491	-0.06	Uknown
Uknown	Uknown	X - 15904	-0.056	Uknown
Uknown	Uknown	X - 23780	0.053	Uknown
Uknown	Uknown	X - 23639	-0.053	Uknown
Uknown	Uknown	X - 24341	0.052	Uknown
Uknown	Uknown	X - 21285	0.05	Uknown
Uknown	Uknown	X - 24455	-0.047	Uknown
Uknown	Uknown	X - 13553	0.041	Uknown
Uknown	Uknown	X - 12026	0.038	Uknown
Uknown	Uknown	X - 24519	0.037	Uknown
Uknown	Uknown	X - 10458	-0.036	Uknown
Uknown	Uknown	X - 23587	0.034	Uknown
Uknown	Uknown	X - 24796	-0.034	Uknown
Uknown	Uknown	X - 12739	0.033	Uknown
Uknown	Uknown	X - 16397	0.033	Uknown
Uknown	Uknown	X - 23678	0.032	Uknown
Uknown	Uknown	X - 22158	-0.031	Uknown
Uknown	Uknown	X - 24361	0.031	Uknown
Uknown	Uknown	X - 11261	0.03	Uknown
Uknown	Uknown	X - 12687	0.028	Uknown
Uknown	Uknown	X - 15461	-0.025	Uknown
Uknown	Uknown	X - 14662	-0.024	Uknown
Uknown	Uknown	X - 24542	-0.023	Uknown
Uknown	Uknown	X - 24669	-0.022	Uknown
Uknown	Uknown	X - 17676	0.021	Uknown
Uknown	Uknown	X - 13844	0.021	Uknown
Uknown	Uknown	X - 12721	0.02	Uknown
Uknown	Uknown	X - 13695	0.02	Uknown
Uknown	Uknown	X - 12821	-0.02	Uknown
Uknown	Uknown	X - 12104	0.018	Uknown
Uknown	Uknown	X - 17354	0.018	Uknown
Uknown	Uknown	X - 24333	0.017	Uknown
Uknown	Uknown	X - 24348	0.016	Uknown
Uknown	Uknown	X - 12704	0.016	Uknown
Uknown	Uknown	X - 17337	0.015	Uknown
Uknown	Uknown	X - 13507	-0.015	Uknown
Uknown	Uknown	X - 12839	0.014	Uknown
Uknown	Uknown	X - 21364	0.014	Uknown
Uknown	Uknown	X - 24359	-0.013	Uknown
Uknown	Uknown	X - 25617	0.012	Uknown
Uknown	Uknown	X - 23644	0.01	Uknown
Uknown	Uknown	X - 24246	-0.01	Uknown
Uknown	Uknown	X - 21845	0.009	Uknown
Uknown	Uknown	X - 21410	0.009	Uknown
Uknown	Uknown	X - 12216	0.009	Uknown
Uknown	Uknown	X - 17365	0.008	Uknown
Uknown	Uknown	X - 17346	-0.005	Uknown

Uknown	Uknown	X - 23787	0.005	Uknown
Uknown	Uknown	X - 22757	0.004	Uknown
Uknown	Uknown	X - 12680	-0.004	Uknown
Uknown	Uknown	X - 21785	0.004	Uknown
Uknown	Uknown	X - 17371	0.003	Uknown
Uknown	Uknown	X - 24543	-0.002	Uknown
Xenobiotics	Benzoate Metabolism	hippurate	0.031	HMDB00714
Xenobiotics	Benzoate Metabolism	4-hydroxymandelate	0.049	HMDB00822
Xenobiotics	Benzoate Metabolism	3-hydroxybenzoate	-0.101	HMDB02466
Xenobiotics	Chemical	glycolate (hydroxyacetate)	-0.049	HMDB00115
Xenobiotics	Chemical	sulfate*	0.145	HMDB01448
Xenobiotics	Chemical	O-sulfo-L-tyrosine	-0.019	HMDB0155722
Xenobiotics	Chemical	3-S-cysteinyl-2-methylpropanoate*	0.004	HMDB30411
Xenobiotics	Chemical	4'-hydroxypropiophenone sulfate	0.014	Uknown
Xenobiotics	Chemical	3-hydroxypyridine glucuronide	-0.001	Uknown
Xenobiotics	Drug - Cardiovascular	4-hydroxycoumarin	0.004	HMDB0003654
Xenobiotics	Drug - Other	S-carboxymethyl-L-cysteine	-0.05	HMDB29415
Xenobiotics	Drug - Respiratory	carboxysuccinate	0.084	Uknown
Xenobiotics	Food Component/Plant	2-isopropylmalate	-0.021	HMDB00402
Xenobiotics	Food Component/Plant	vanillate	-0.003	HMDB00484
Xenobiotics	Food Component/Plant	levulinate (4-oxovalerate)	-0.012	HMDB00720
Xenobiotics	Food Component/Plant	quinate	0.027	HMDB03072
Xenobiotics	Food Component/Plant	cinnamoylglycine	0.057	HMDB11621
Xenobiotics	Food Component/Plant	ferulic acid 4-sulfate	0.062	HMDB29200
Xenobiotics	Food Component/Plant	acesulfame	0.006	HMDB33585
Xenobiotics	Food Component/Plant	sulfate of piperine metabolite C16H19NO4 (3)*	0.076	Uknown
Xenobiotics	Food Component/Plant	sulfate of piperine metabolite C16H19NO3 (2)*	0.07	Uknown
Xenobiotics	Food Component/Plant	sulforaphane-cysteine	-0.067	Uknown
Xenobiotics	Food Component/Plant	ethyl alpha-glucopyranoside	0.066	Uknown
Xenobiotics	Food Component/Plant	sulfate of piperine metabolite C16H19NO3 (1)*	0.056	Uknown
Xenobiotics	Food Component/Plant	sulfate of piperine metabolite C18H21NO3 (2)*	0.036	Uknown
Xenobiotics	Food Component/Plant	isoeugenol sulfate	0.034	Uknown
Xenobiotics	Food Component/Plant	sulfate of piperine metabolite C18H21NO3 (1)*	0.029	Uknown
Xenobiotics	Food Component/Plant	sulfate of piperine metabolite C16H19NO3 (3)*	0.016	Uknown
Xenobiotics	Food Component/Plant	sulfate of piperine metabolite C16H19NO4 (4)*	0.015	Uknown
Xenobiotics	Food Component/Plant	eugenol sulfate	0.009	Uknown
Xenobiotics	Food Component/Plant	sulfate of piperine metabolite C18H21NO3 (3)*	0.007	Uknown
Xenobiotics	Food Component/Plant	sulfate of piperine metabolite C16H19NO4 (2)*	0.006	Uknown
Xenobiotics	Food Component/Plant	sulfate of piperine metabolite C15H17NO3 (2)*	0.004	Uknown
Xenobiotics	Food Component/Plant	sulfate of piperine metabolite C15H17NO3 (1)*	0.002	Uknown

Xenobiotics	Xanthine Metabolism	3-methylurate*	-0.057	HMDB0001970
Xenobiotics	Xanthine Metabolism	3-methylxanthine	-0.11	HMDB01886
Xenobiotics	Xanthine Metabolism	3,7-dimethylurate	-0.009	HMDB01982
Xenobiotics	Xanthine Metabolism	7-methylxanthine	-0.091	HMDB01991
Xenobiotics	Xanthine Metabolism	theobromine	-0.131	HMDB02825
Xenobiotics	Xanthine Metabolism	1-methylurate	0.005	HMDB03099
Xenobiotics	Xanthine Metabolism	7-methylurate	-0.015	HMDB11107

PCM, Partially Characterized Molecules.

*Indicates a compound that has not been confirmed based on authentic chemical standard, but Metabolon are confident in its identity. The structural identities of ‘X-’ followed by a number (e.g., X - 11372) are unknown.

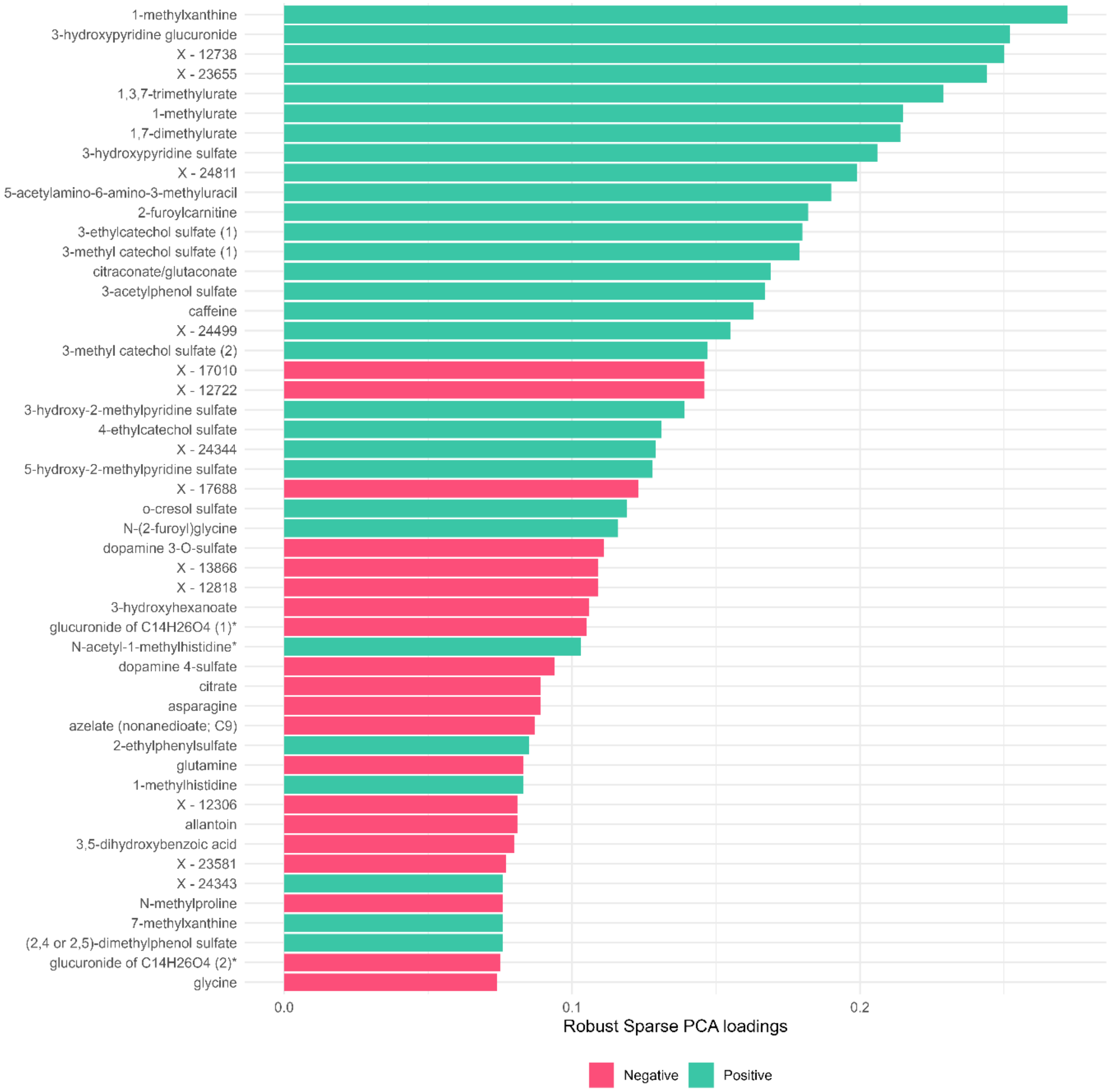

Supplemental Figure 3: Top 50 metabolites by absolute weight loads in MP9 in adolescent urine samples.

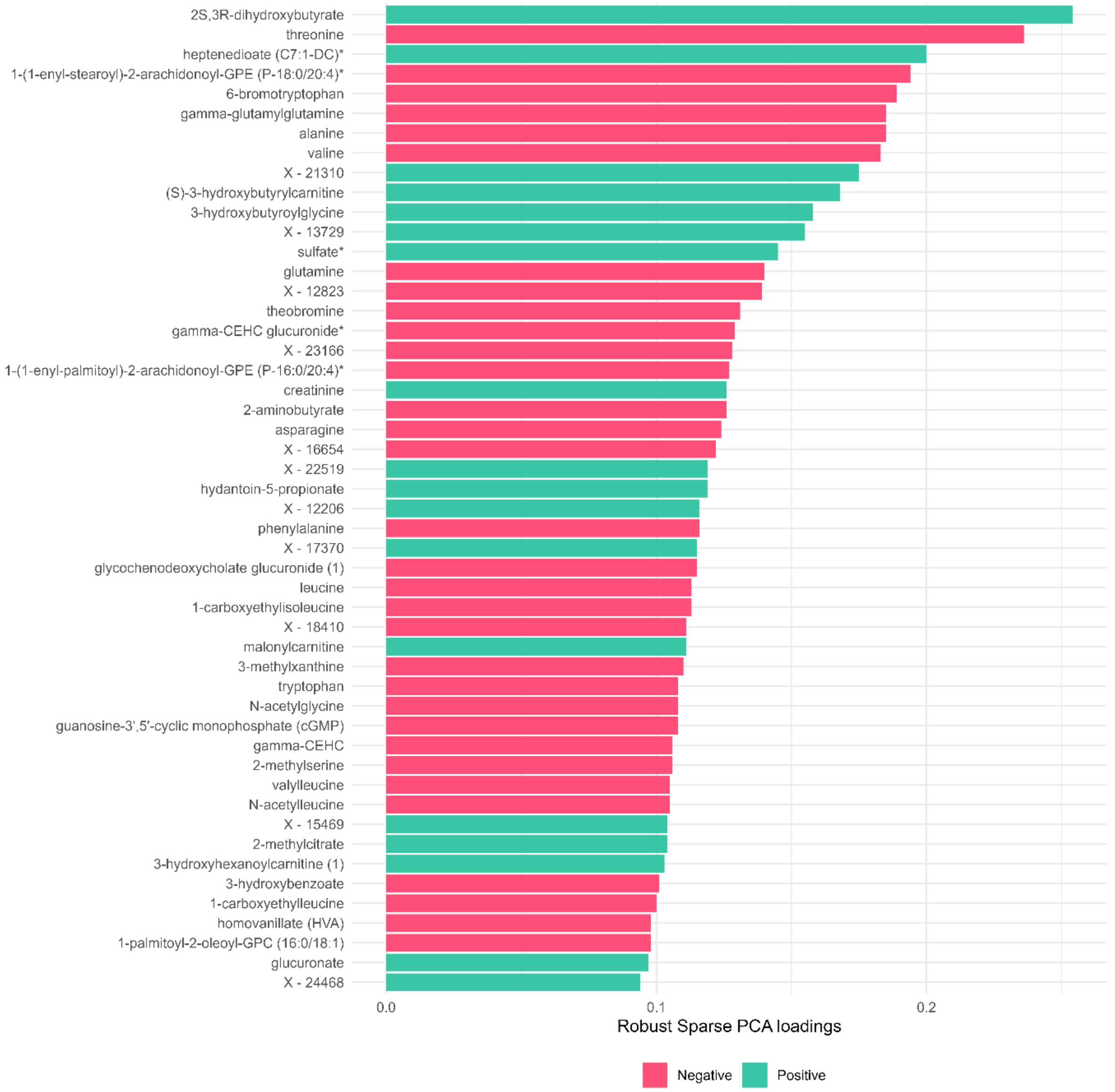

Supplemental Figure 4: Top 50 metabolites by absolute weight loads in MP7 in adolescent urine samples.

Supplemental Table 6. Regression estimates of the associations between UPF intake and plasma metabolite patterns in young adults

Metabolite pattern	Model 1, β (95% CI)	Model 2, β (95% CI)	Model 3, β (95% CI)
**MP1**	**-0.021 (-0.100, 0.058)**	**-0.081 (-0.134, -0.029)[1]**	-0.064 (-0.119, -0.010)
MP2	0.051 (-0.021, 0.122)	0.058 (-0.017, 0.132)	0.026 (-0.048, 0.100)
MP3	0.044 (-0.006, 0.095)	0.042 (-0.008, 0.092)	0.010 (-0.040, 0.060)
MP4	-0.005 (-0.051, 0.041)	-0.023 (-0.070, 0.024)	-0.053 (-0.096, -0.009)
MP5	0.012 (-0.032, 0.055)	0.013 (-0.031, 0.058)	0.007 (-0.040, 0.053)
**MP6**	**0.047 (0.005, 0.088)**	**0.063 (0.023, 0.104)[1]**	0.049 (0.008, 0.091)
MP7	0.033 (-0.007, 0.074)	0.038 (-0.005, 0.080)	0.027 (-0.017, 0.071)
**MP8**	**0.065 (0.026, 0.105)[1]**	**0.072 (0.031, 0.113)[1]**	**0.074 (0.031, 0.117)[1]**
MP9	-0.001 (-0.037, 0.035)	0.001 (-0.037, 0.039)	0.002 (-0.037, 0.041)
MP10	-0.006 (-0.041, 0.029)	0.005 (-0.031, 0.041)	0.010 (-0.027, 0.048)
MP11	-0.025 (-0.060, 0.010)	-0.024 (-0.061, 0.013)	-0.029 (-0.068, 0.009)
MP12	0.035 (0.001, 0.069)	0.024 (-0.011, 0.059)	0.019 (-0.017, 0.056)
MP13	-0.010 (-0.044, 0.024)	-0.006 (-0.040, 0.029)	0.001 (-0.035, 0.037)
MP14	0.002 (-0.030, 0.033)	0.002 (-0.030, 0.033)	0.001 (-0.031, 0.033)
MP15	0.008 (-0.022, 0.039)	0.008 (-0.024, 0.040)	0.005 (-0.028, 0.038)
MP16	0.003 (-0.029, 0.035)	0.008 (-0.025, 0.041)	-0.001 (-0.035, 0.033)
**MP17**	**-0.040 (-0.071, -0.010)**	**-0.052 (-0.083, -0.021)[1]**	-0.038 (-0.070, -0.007)
MP18	0.001 (-0.027, 0.030)	-0.001 (-0.030, 0.029)	-0.000 (-0.030, 0.030)
MP19	-0.011 (-0.040, 0.017)	-0.011 (-0.040, 0.019)	-0.006 (-0.036, 0.025)

[1]statistical significance (FDR q-value <0.05).
Model 1: unadjusted.
Model 2: adjusted for age, sex, body mass index, energy intake, number of dietary assessments, and time difference between dietary assessment and blood draw.
Model 3: Adjustments model 2 and physical activity, alcohol and smoking status.
Abbreviations: β, regression estimate; CI, confidence intervals; FDR, false discovery rate according to Benjamini-Hochberg; MP, metabolite pattern.

Supplemental Table 7: Metabolites with non-zero loadings in plasma MP8.

super_pathway	sub_pathway	metabolite	Loadings	Hmdb
Amino Acid	Glycine, Serine and Threonine Metabolism	glycine	-0.119	HMDB00123
Amino Acid	Methionine, Cysteine, SAM and Taurine Metabolism	N-acetylmethionine	0.095	HMDB11745
Amino Acid	Urea cycle; Arginine and Proline Metabolism	N-methylproline	-0.086	Uknown
Amino Acid	Glutamate Metabolism	4-hydroxyglutamate	0.08	HMDB01344
Amino Acid	Urea cycle; Arginine and Proline Metabolism	proline	-0.073	HMDB00162
Amino Acid	Alanine and Aspartate Metabolism	aspartate	0.07	HMDB00191
Amino Acid	Histidine Metabolism	1-methylhistidine	0.069	HMDB00001
Amino Acid	Histidine Metabolism	formiminoglutamate	0.064	HMDB00854
Amino Acid	Tyrosine Metabolism	phenol sulfate	-0.061	HMDB60015
Amino Acid	Urea cycle; Arginine and Proline Metabolism	homocitrulline	0.054	HMDB00679
Amino Acid	Creatine Metabolism	guanidinoacetate	-0.052	HMDB00128
Amino Acid	Leucine, Isoleucine and Valine Metabolism	3-hydroxy-2-ethylpropionate	0.051	HMDB00396
Amino Acid	Leucine, Isoleucine and Valine Metabolism	ethylmalonate	0.049	HMDB00622
Amino Acid	Methionine, Cysteine, SAM and Taurine Metabolism	cysteine	-0.046	HMDB00574
Amino Acid	Lysine Metabolism	N6,N6,N6-trimethyllysine	0.044	HMDB01325
Amino Acid	Leucine, Isoleucine and Valine Metabolism	N-acetylvaline	0.038	HMDB11757
Amino Acid	Tyrosine Metabolism	tyrosine	-0.031	HMDB00158
Amino Acid	Glutathione Metabolism	2-aminobutyrate	0.031	HMDB00650
Amino Acid	Leucine, Isoleucine and Valine Metabolism	2-hydroxy-3-methylvalerate	0.021	HMDB00317
Amino Acid	Lysine Metabolism	6-oxopiperidine-2-carboxylate	0.019	HMDB61705
Amino Acid	Tyrosine Metabolism	gentisate	-0.019	HMDB00152
Amino Acid	Methionine, Cysteine, SAM and Taurine Metabolism	cysteine s-sulfate	0.011	HMDB00731
Amino Acid	Urea cycle; Arginine and Proline Metabolism	hydroxyproline	0.009	HMDB00725
Amino Acid	Methionine, Cysteine, SAM and Taurine Metabolism	S-adenosylhomocysteine (SAH)	0.008	HMDB00939
Amino Acid	Histidine Metabolism	3-methylhistidine	0.007	HMDB00479
Amino Acid	Leucine, Isoleucine and Valine Metabolism	beta-hydroxyisovalerate	0.006	HMDB00754
Amino Acid	Alanine and Aspartate Metabolism	N-acetylalanine	0.005	HMDB00766
Amino Acid	Urea cycle; Arginine and Proline Metabolism	homoarginine	0.003	HMDB00670
Carbohydrate	Pentose Metabolism	arabonate/xylonate	-0.108	Uknown
Carbohydrate	Pentose Metabolism	ribonate	-0.006	HMDB00867
Cofactors and Vitamins	Vitamin A Metabolism	retinal	0.063	HMDB01358

Cofactors and Vitamins	Hemoglobin and Porphyrin Metabolism	heme	0.045	HMDB03178
Cofactors and Vitamins	Ascorbate and Aldarate Metabolism	oxalate (ethanedioate)	-0.042	HMDB02329
Cofactors and Vitamins	Vitamin A Metabolism	beta-cryptoxanthin	-0.034	HMDB33844
Cofactors and Vitamins	Pantothenate and CoA Metabolism	pantothenate (Vitamin B5)	-0.031	HMDB00210
Cofactors and Vitamins	Vitamin A Metabolism	carotene diol (1)	-0.024	Unknown
Cofactors and Vitamins	Hemoglobin and Porphyrin Metabolism	bilirubin	-0.01	HMDB00054
Cofactors and Vitamins	Nicotinate and Nicotinamide Metabolism	1-methylnicotinamide	0.004	HMDB00699
Energy	TCA Cycle	fumarate	-0.055	HMDB00134
Lipid	Secondary Bile Acid Metabolism	taurolithocholate 3-sulfate	0.184	HMDB02580
Lipid	Secondary Bile Acid Metabolism	taurocholenate sulfate*	0.173	Unknown
Lipid	Fatty Acid, Dicarboxylate	eicosanedioate (C20-DC)	0.17	Unknown
Lipid	Fatty Acid, Dicarboxylate	docosadioate (C22-DC)	0.135	Unknown
Lipid	Fatty Acid Metabolism (Acyl Choline)	palmitoylcholine	-0.126	Unknown
Lipid	Fatty Acid Metabolism (Acyl Choline)	stearoylcholine*	-0.119	Unknown
Lipid	Fatty Acid Metabolism (Acyl Choline)	oleoylcholine	-0.118	Unknown
Lipid	Fatty Acid Metabolism (Acyl Carnitine, Hydroxy)	3-hydroxyoleoylcarnitine	0.113	Unknown
Lipid	Fatty Acid, Dihydroxy	2S,3R-dihydroxybutyrate	0.11	HMDB02453
Lipid	Fatty Acid, Monohydroxy	16-hydroxypalmitate	-0.107	HMDB06294
Lipid	Androgenic Steroids	androstenediol (3alpha, 17alpha) monosulfate (3)	-0.098	Unknown
Lipid	Fatty Acid, Dicarboxylate	hexadecenedioate (C16:1-DC)*	0.095	Unknown
Lipid	Androgenic Steroids	5alpha-androstan-3alpha,17alpha-diol monosulfate	-0.093	Unknown
Lipid	Fatty Acid, Monohydroxy	3-hydroxyoctanoate	0.092	HMDB01954
Lipid	Androgenic Steroids	androsterone sulfate	-0.089	HMDB02759
Lipid	Secondary Bile Acid Metabolism	taurochenodeoxycholic acid 3-sulfate	0.086	HMDB02486
Lipid	Sterol	7-HOCA	-0.081	HMDB12458
Lipid	Fatty Acid Metabolism (Acyl Carnitine, Monounsaturated)	eicosenoylcarnitine (C20:1)*	0.081	Unknown
Lipid	Inositol Metabolism	myo-inositol	-0.078	HMDB00211
Lipid	Fatty Acid Metabolism (Acyl Choline)	linoleoylcholine*	-0.074	Unknown
Lipid	Long Chain Polyunsaturated Fatty Acid (n3 and n6)	mead acid (20:3n9)	-0.071	HMDB10378
Lipid	Fatty Acid, Monohydroxy	3-hydroxyhexanoate	0.07	Unknown
Lipid	Medium Chain Fatty Acid	caprate (10:0)	-0.069	HMDB00511

Lipid	Long Chain Polyunsaturated Fatty Acid (n3 and n6)	docosapentaenoate (DPA; 22:5n3)	-0.068	HMDB06528
Lipid	Fatty Acid, Dicarboxylate	branched chain 14:0 dicarboxylic acid	-0.067	Unknown
Lipid	Fatty Acid, Dicarboxylate	decadienedioic acid (C10:2-DC)	0.067	Unknown
Lipid	Medium Chain Fatty Acid	laurate (12:0)	-0.066	HMDB00638
Lipid	Long Chain Saturated Fatty Acid	nonadecanoate (19:0)	-0.064	HMDB00772
Lipid	Fatty Acid, Monohydroxy	13-HODE + 9-HODE	-0.062	Unknown
Lipid	Fatty Acid Metabolism (Acyl Choline)	arachidonoylcholine	-0.061	Unknown
Lipid	Progestin Steroids	pregnanolone/allopregnanolone sulfate	-0.059	Unknown
Lipid	Androgenic Steroids	androstenediol (3alpha, 17alpha) monosulfate (2)	-0.059	Unknown
Lipid	Fatty Acid Metabolism (Acyl Carnitine, Monounsaturated)	myristoleoylcarnitine (C14:1)*	0.056	Unknown
Lipid	Fatty Acid, Branched	(16 or 17)-methylstearate (a19:0 or i19:0)	-0.055	HMDB37397
Lipid	Fatty Acid Metabolism (Acyl Glycine)	picolinoylglycine	0.054	HMDB59766
Lipid	Long Chain Polyunsaturated Fatty Acid (n3 and n6)	eicosapentaenoate (EPA; 20:5n3)	-0.053	HMDB01999
Lipid	Primary Bile Acid Metabolism	taurochenodeoxycholate	0.053	HMDB00951
Lipid	Fatty Acid, Dicarboxylate	4-hydroxy-2-oxoglutaric acid	-0.053	HMDB02070
Lipid	Long Chain Saturated Fatty Acid	pentadecanoate (15:0)	-0.052	HMDB00826
Lipid	Long Chain Polyunsaturated Fatty Acid (n3 and n6)	dihomolinolenate (20:3n3 or 3n6)	-0.049	HMDB02925
Lipid	Long Chain Polyunsaturated Fatty Acid (n3 and n6)	docosahexaenoate (DHA; 22:6n3)	-0.048	HMDB02183
Lipid	Fatty Acid Metabolism (Acyl Choline)	docosahexaenoylcholine	-0.047	Unknown
Lipid	Progestin Steroids	5alpha-pregnan-3beta,20beta-diol monosulfate (1)	-0.046	Unknown
Lipid	Fatty Acid, Monohydroxy	2-hydroxystearate	-0.045	Unknown
Lipid	Fatty Acid, Branched	pristanate	-0.043	HMDB00795
Lipid	Fatty Acid Metabolism (Acyl Carnitine, Monounsaturated)	nervonoylcarnitine (C24:1)*	0.043	Unknown
Lipid	Fatty Acid Metabolism (Acyl Choline)	dihomo-linolenoyl-choline	-0.041	Unknown
Lipid	Fatty Acid, Branched	(14 or 15)-methylpalmitate (a17:0 or i17:0)	-0.041	Unknown
Lipid	Secondary Bile Acid Metabolism	ursodeoxycholate	-0.036	HMDB00946
Lipid	Long Chain Saturated Fatty Acid	margarate (17:0)	-0.034	HMDB02259
Lipid	Fatty Acid, Monohydroxy	2-hydroxypalmitate	-0.032	HMDB31057
Lipid	Pregnenolone Steroids	21-hydroxypregnenolone monosulfate (1)	-0.03	Unknown
Lipid	Fatty Acid, Dicarboxylate	eicosenedioate (C20:1-DC)*	0.025	Unknown

Lipid	Progestin Steroids	5alpha-pregnan-3beta,20alpha-diol monosulfate (2)	-0.025	Unknown
Lipid	Pregnenolone Steroids	pregnenolone sulfate	-0.025	HMDB00774
Lipid	Lysophospholipid	1-palmitoyl-GPG (16:0)*	-0.024	Unknown
Lipid	Secondary Bile Acid Metabolism	taurodeoxycholic acid 3-sulfate	0.022	Unknown
Lipid	Fatty Acid, Dicarboxylate	glutarate (C5-DC)	-0.022	HMDB00661
Lipid	Fatty Acid, Monohydroxy	2-hydroxynervonate*	0.021	Unknown
Lipid	Fatty Acid Metabolism (Acyl Glycine)	N-palmitoylglycine	-0.019	HMDB13034
Lipid	Androgenic Steroids	11beta-hydroxyandrosterone glucuronide	-0.019	Unknown
Lipid	Long Chain Polyunsaturated Fatty Acid (n3 and n6)	docosapentaenoate (n6 DPA; 22:5n6)	-0.019	HMDB01976
Lipid	Androgenic Steroids	epiandrosterone sulfate	-0.018	Unknown
Lipid	Fatty Acid Metabolism (also BCAA Metabolism)	methylmalonate (MMA)	0.018	HMDB00202
Lipid	Fatty Acid Metabolism (also BCAA Metabolism)	butyrylcarnitine (C4)	0.017	HMDB02013
Lipid	Androgenic Steroids	5alpha-androstan-3alpha,17beta-diol monosulfate (1)	-0.017	Unknown
Lipid	Corticosteroids	cortisol	0.017	HMDB00063
Lipid	Long Chain Saturated Fatty Acid	myristate (14:0)	-0.017	HMDB00806
Lipid	Fatty Acid Metabolism (also BCAA Metabolism)	propionylglycine (C3)	-0.017	HMDB00783
Lipid	Fatty Acid Metabolism (Acyl Carnitine, Monounsaturated)	cis-4-decenoylcarnitine (C10:1)	0.016	Unknown
Lipid	Fatty Acid, Amide	pentadecanamide (15:0)*	0.014	Unknown
Lipid	Fatty Acid, Amino	2-aminooctanoate	0.014	HMDB00991
Lipid	Fatty Acid, Branched	branched-chain fatty acid 18:0 (2)	0.013	Unknown
Lipid	Long Chain Polyunsaturated Fatty Acid (n3 and n6)	arachidonate (20:4n6)	-0.01	HMDB01043
Lipid	Primary Bile Acid Metabolism	taurocholate	0.01	HMDB00036
Lipid	Secondary Bile Acid Metabolism	isoursodeoxycholate	-0.009	HMDB00686
Lipid	Fatty Acid Metabolism (Acyl Carnitine, Monounsaturated)	palmitoleoylcarnitine (C16:1)*	0.007	Unknown
Lipid	Progestin Steroids	pregnanediol-3-glucuronide	-0.006	HMDB10318
Lipid	Fatty Acid Metabolism (Acyl Carnitine, Long Chain Saturated)	margaroylcarnitine (C17)*	-0.006	HMDB06210
Lipid	Fatty Acid, Dicarboxylate	octadecadienedioate (C18:2-DC)*	0.006	Unknown
Lipid	Androgenic Steroids	dehydroepiandrosterone sulfate (DHEA-S)	-0.006	HMDB01032
Lipid	Endocannabinoid	N-palmitoylserine	-0.005	Unknown
Lipid	Primary Bile Acid Metabolism	cholate	-0.005	HMDB00619

Lipid	Fatty Acid, Dihydroxy	3,4-dihydroxybutyrate	0.003	Unknown
Lipid	Fatty Acid Metabolism (Acyl Carnitine, Medium Chain)	laurylcarnitine (C12)	0.002	HMDB02250
Lipid	Fatty Acid, Monohydroxy	3-hydroxyoleate*	0.001	Unknown
Nucleotide	Pyrimidine Metabolism, Uracil containing	2'-O-methyluridine	0.007	Unknown
Partially Characterized Molecules	Partially Characterized Molecules	glycine conjugate of $C_{10}H_{14}O_2$ (1)*	0.12	Unknown
Partially Characterized Molecules	Partially Characterized Molecules	pentose acid*	-0.065	Unknown
Partially Characterized Molecules	Partially Characterized Molecules	glutamine conjugate of $C_6H_{10}O_2$ (2)*	0.041	Unknown
Peptide	Acetylated Peptides	phenylacetylglutamine	0.031	HMDB06344
Peptide	Gamma-glutamyl Amino Acid	gamma-glutamylglycine	-0.015	HMDB11667
Peptide	Gamma-glutamyl Amino Acid	gamma-glutamylglutamine	-0.001	HMDB11738
Unknown	Unknown	X - 21319	0.221	Unknown
Unknown	Unknown	X - 15486	0.209	Unknown
Unknown	Unknown	X - 16944	0.205	Unknown
Unknown	Unknown	X - 11261	0.189	Unknown
Unknown	Unknown	X - 11478	0.179	Unknown
Unknown	Unknown	X - 11880	0.165	Unknown
Unknown	Unknown	X - 14939	0.164	Unknown
Unknown	Unknown	X - 16935	0.157	Unknown
Unknown	Unknown	X - 18921	0.15	Unknown
Unknown	Unknown	X - 25433	0.147	Unknown
Unknown	Unknown	X - 16580	0.146	Unknown
Unknown	Unknown	X - 21829	0.144	Unknown
Unknown	Unknown	X - 21736	0.132	Unknown
Unknown	Unknown	X - 11308	0.13	Unknown
Unknown	Unknown	X - 23680	0.128	Unknown
Unknown	Unknown	X - 11372	0.118	Unknown
Unknown	Unknown	X - 11315	-0.099	Unknown
Unknown	Unknown	X - 12216	0.082	Unknown
Unknown	Unknown	X - 12101	0.081	Unknown
Unknown	Unknown	X - 24951	0.081	Unknown
Unknown	Unknown	X - 18922	0.076	Unknown
Unknown	Unknown	X - 17335	0.073	Unknown
Unknown	Unknown	X - 17357	-0.066	Unknown
Unknown	Unknown	X - 17653	0.064	Unknown
Unknown	Unknown	X - 12844	-0.049	Unknown
Unknown	Unknown	X - 12096	-0.048	Unknown
Unknown	Unknown	X - 18886	0.047	Unknown
Unknown	Unknown	X - 18899	0.046	Unknown
Unknown	Unknown	X - 21339	0.042	Unknown
Unknown	Unknown	X - 12680	-0.04	Unknown
Unknown	Unknown	X - 17612	0.038	Unknown
Unknown	Unknown	X - 21467	-0.037	Unknown
Unknown	Unknown	X - 12112	0.035	Unknown
Unknown	Unknown	X - 11795	-0.03	Unknown

Unknown	Unknown	X - 23639	-0.021	Unknown
Unknown	Unknown	X - 15503	0.02	Unknown
Unknown	Unknown	X - 12015	-0.02	Unknown
Unknown	Unknown	X - 24947	-0.014	Unknown
Unknown	Unknown	X - 24435	0.01	Unknown
Unknown	Unknown	X - 11442	-0.005	Unknown
Unknown	Unknown	X - 25828	-0.004	Unknown
Unknown	Unknown	X - 25810	-0.004	Unknown
Unknown	Unknown	X - 21607	0.004	Unknown
Unknown	Unknown	X - 17340	-0.003	Unknown
Unknown	Unknown	X - 11441	-0.001	Unknown
Xenobiotics	Benzoate Metabolism	3-methoxycatechol sulfate (2)	-0.174	Unknown
Xenobiotics	Xanthine Metabolism	caffeine	0.162	HMDB01847
Xenobiotics	Food Component/Plant	stachydrine	-0.146	HMDB04827
Xenobiotics	Food Component/Plant	3-hydroxystachydrine*	-0.139	Unknown
Xenobiotics	Xanthine Metabolism	5-acetylamino-6-formylamino-3-methyluracil	0.125	HMDB11105
Xenobiotics	Food Component/Plant	ethyl beta-glucopyranoside	-0.125	Unknown
Xenobiotics	Xanthine Metabolism	theophylline	0.122	HMDB01889
Xenobiotics	Food Component/Plant	methyl glucopyranoside (alpha + beta)	-0.119	Unknown
Xenobiotics	Xanthine Metabolism	paraxanthine	0.112	HMDB01860
Xenobiotics	Food Component/Plant	4-allylphenol sulfate	-0.112	Unknown
Xenobiotics	Xanthine Metabolism	1,7-dimethylurate	0.111	HMDB11103
Xenobiotics	Drug - Topical Agents	2,6-dihydroxybenzoic acid	-0.096	HMDB13676
Xenobiotics	Xanthine Metabolism	5-acetylamino-6-amino-3-methyluracil	0.095	HMDB04400
Xenobiotics	Xanthine Metabolism	1-methylxanthine	0.079	HMDB10738
Xenobiotics	Food Component/Plant	sulfate of piperine metabolite C16H19NO3 (2)*	0.078	Unknown
Xenobiotics	Benzoate Metabolism	4-hydroxyhippurate	-0.078	HMDB13678
Xenobiotics	Chemical	2-aminophenol sulfate	-0.078	HMDB61116
Xenobiotics	Food Component/Plant	glucuronide of piperine metabolite C17H21NO3 (5)*	0.078	Unknown
Xenobiotics	Food Component/Plant	glucuronide of piperine metabolite C17H21NO3 (4)*	0.075	Unknown
Xenobiotics	Food Component/Plant	sulfate of piperine metabolite C18H21NO3 (1)*	0.074	Unknown
Xenobiotics	Drug - Topical Agents	hydroquinone sulfate	-0.073	HMDB02434
Xenobiotics	Food Component/Plant	sulfate of piperine metabolite C16H19NO3 (3)*	0.068	Unknown
Xenobiotics	Food Component/Plant	piperine	0.057	HMDB29377
Xenobiotics	Food Component/Plant	sulfate of piperine metabolite C18H21NO3 (3)*	0.049	Unknown
Xenobiotics	Food Component/Plant	tartronate (hydroxymalonate)	-0.046	HMDB35227
Xenobiotics	Food Component/Plant	glucuronide of piperine metabolite C17H21NO3 (3)*	0.045	Unknown
Xenobiotics	Benzoate Metabolism	3-hydroxyhippurate	-0.042	HMDB06116
Xenobiotics	Food Component/Plant	erythritol	-0.042	HMDB02994
Xenobiotics	Food Component/Plant	homostachydrine*	-0.039	HMDB33433

Xenobiotics	Benzoate Metabolism	p-cresol sulfate	0.036	HMDB11635
Xenobiotics	Chemical	3,5-dichloro-2,6-dihydroxybenzoic acid	0.034	Unknown
Xenobiotics	Benzoate Metabolism	3-methoxycatechol sulfate (1)	-0.033	Unknown
Xenobiotics	Food Component/Plant	phytanate	-0.024	HMDB00801
Xenobiotics	Food Component/Plant	ergothioneine	-0.016	HMDB03045
Xenobiotics	Chemical	sulfate*	0.012	HMDB01448
Xenobiotics	Benzoate Metabolism	3-(3-hydroxyphenyl) propionate	-0.011	HMDB00375
Xenobiotics	Benzoate Metabolism	guaiacol sulfate	-0.009	HMDB60013
Xenobiotics	Food Component/Plant	3,4-methyleneheptanoate	0.005	Unknown
Xenobiotics	Chemical	4-chlorobenzoic acid	0.002	Unknown

*Indicates a compound that has not been confirmed based on authentic chemical standard, but Metabolon are confident in its identity. The structural identities of 'X-' followed by a number (e.g., X - 11372) are unknown.

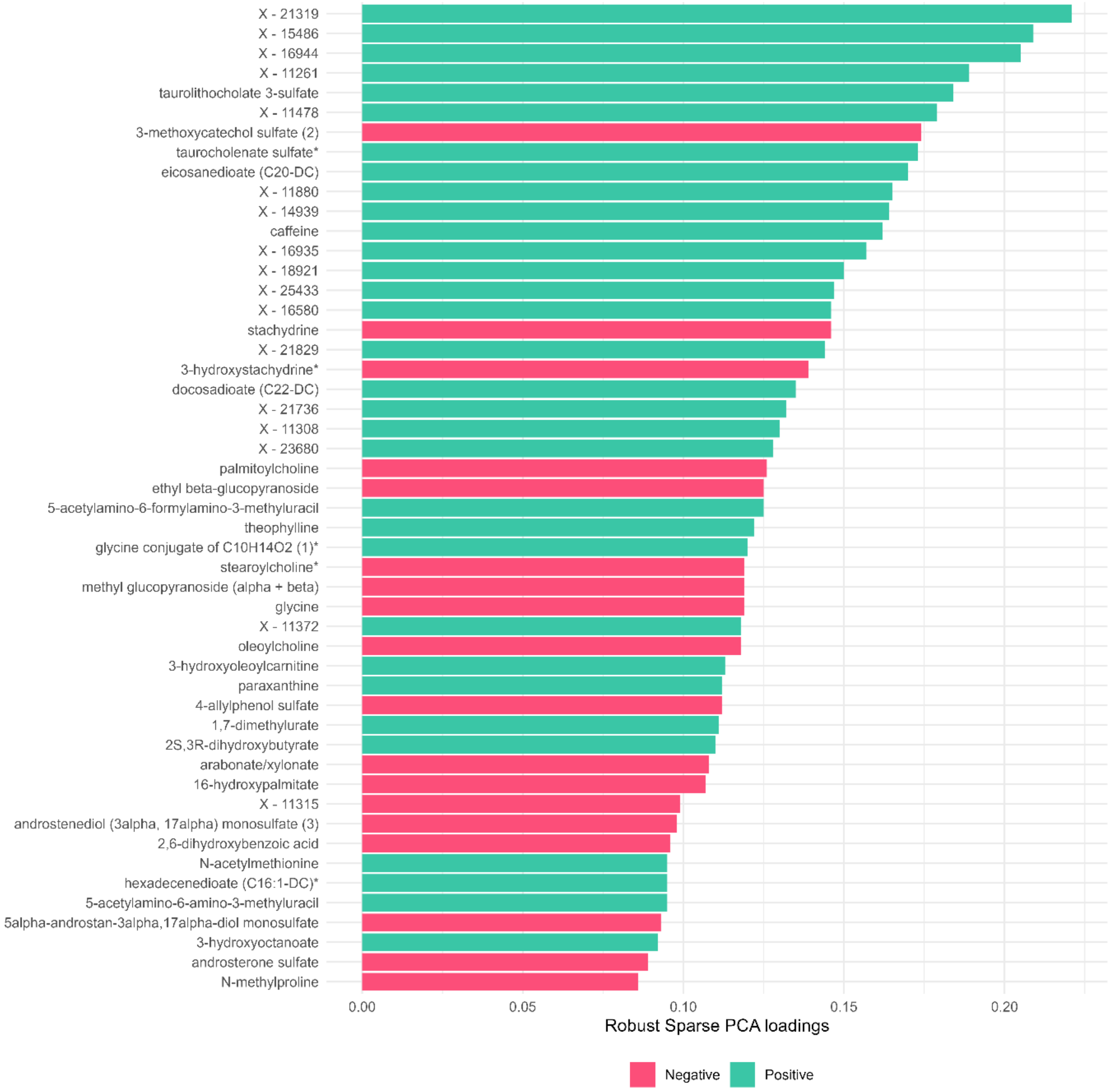

Supplemental Figure 5: Top 50 metabolites by absolute weight loads in MP8 in young adult plasma samples.

Supplemental Table 8. Regression estimates of the associations of absolute and energy-based UPF intake with urine metabolites

Metabolite	UPF (absolute intake, g/day)		UPF (% TEI, Kcal)	
	β (95%, CI)	FDR q-value	β (95%, CI)	FDR q-value
X - 17679	0.0008 (0.0006, 0.0010)	0.0000	0.014 (0.006, 0.021)	0.0020
glucuronide of C10H14O2 (2)*	0.0004 (0.0002, 0.0006)	0.0006	0.008 (-0.000, 0.015)	0.0715
glucuronide of C10H18O2 (1)*	0.0004 (0.0002, 0.0006)	0.0006	0.008 (0.001, 0.016)	0.0444
N,N-dimethylalanine	0.0004 (0.0002, 0.0006)	0.0006	0.011 (0.004, 0.019)	0.0072
glucuronide of C10H18O2 (7)*	0.0004 (0.0002, 0.0006)	0.0006	0.008 (0.000, 0.016)	0.0551
X - 11478	0.0004 (0.0002, 0.0006)	0.0006	0.009 (0.001, 0.016)	0.0402
X - 21807	-0.0004 (-0.0006, -0.0002)	0.0007	-0.015 (-0.022, -0.007)	0.0006
X - 12818	-0.0004 (-0.0006, -0.0002)	0.0007	-0.017 (-0.025, -0.010)	0.0001
glucuronide of C10H18O2 (8)*	0.0004 (0.0002, 0.0006)	0.0007	0.007 (-0.001, 0.015)	0.0874
indoxyl glucuronide	0.0004 (0.0002, 0.0006)	0.0008	0.010 (0.003, 0.018)	0.0107
caffeic acid sulfate	-0.0004 (-0.0006, -0.0002)	0.0008	-0.019 (-0.026, -0.012)	0.0000
3-methyladipate	-0.0003 (-0.0005, -0.0002)	0.0020	-0.012 (-0.019, -0.005)	0.0043
allantoic acid	-0.0004 (-0.0006, -0.0002)	0.0021	-0.007 (-0.015, 0.001)	0.0817
glucuronide of C10H18O2 (9)*	0.0004 (0.0002, 0.0006)	0.0022	0.005 (-0.003, 0.012)	0.2655
tiglyl carnitine (C5)	-0.0003 (-0.0005, -0.0001)	0.0022	-0.011 (-0.018, -0.004)	0.0046
3-hydroxysebacate	-0.0003 (-0.0005, -0.0001)	0.0022	-0.000 (-0.008, 0.007)	0.9582
1-methylhistamine	0.0003 (0.0001, 0.0005)	0.0023	0.013 (0.005, 0.020)	0.0032
1,6-anhydroglucose	0.0004 (0.0001, 0.0006)	0.0023	0.014 (0.007, 0.022)	0.0012
3,5-dihydroxybenzoic acid	-0.0003 (-0.0005, -0.0001)	0.0025	-0.016 (-0.023, -0.008)	0.0003
X - 13844	-0.0003 (-0.0005, -0.0001)	0.0030	-0.013 (-0.020, -0.006)	0.0023
4-methoxyphenol sulfate	-0.0003 (-0.0005, -0.0001)	0.0044	-0.017 (-0.024, -0.009)	0.0001
X - 19497	0.0003 (0.0001, 0.0005)	0.0045	0.012 (0.005, 0.020)	0.0042
hydroquinone sulfate	-0.0003 (-0.0005, -0.0001)	0.0045	-0.009 (-0.016, -0.001)	0.0340
allantoin	-0.0003 (-0.0005, -0.0001)	0.0048	-0.006 (-0.014, 0.001)	0.1213
5-acetylamino-6-amino-3-methyluracil	0.0003 (0.0001, 0.0005)	0.0048	0.009 (0.001, 0.016)	0.0359
X - 17358	0.0003 (0.0001, 0.0005)	0.0056	0.005 (-0.003, 0.012)	0.2428
hydroxy-N6,N6,N6-trimethyllysine*	0.0003 (0.0001, 0.0005)	0.0066	0.014 (0.006, 0.021)	0.0011
2-acetamidophenol sulfate	-0.0003 (-0.0005, -0.0001)	0.0066	-0.011 (-0.018, -0.003)	0.0107

X - 13695	-0.0003 (-0.0005, -0.0001)	0.0066	-0.015 (-0.023, -0.007)	0.0006
X - 16087	-0.0003 (-0.0005, -0.0001)	0.0066	-0.004 (-0.011, 0.003)	0.3061
syringic acid	-0.0003 (-0.0005, -0.0001)	0.0110	-0.015 (-0.022, -0.007)	0.0006
1-methylguanine	0.0003 (0.0001, 0.0005)	0.0115	0.009 (0.001, 0.016)	0.0298
X - 23459	-0.0003 (-0.0005, -0.0001)	0.0115	-0.010 (-0.017, -0.002)	0.0223
X - 24345	0.0003 (0.0001, 0.0005)	0.0129	0.010 (0.002, 0.017)	0.0208
5-hydroxy-2-methylpyridine sulfate	0.0003 (0.0001, 0.0005)	0.0151	0.005 (-0.002, 0.013)	0.2016
heptenedioate (C7:1-DC)*	-0.0003 (-0.0004, -0.0001)	0.0161	-0.009 (-0.016, -0.001)	0.0298
dihydroferulic acid sulfate	-0.0003 (-0.0005, -0.0001)	0.0164	-0.017 (-0.025, -0.010)	0.0001
o-cresol sulfate	0.0003 (0.0001, 0.0004)	0.0171	0.010 (0.002, 0.017)	0.0162
X - 17676	-0.0002 (-0.0004, -0.0001)	0.0198	-0.002 (-0.009, 0.005)	0.5999
ferulic acid 4-sulfate	-0.0003 (-0.0005, -0.0001)	0.0198	-0.020 (-0.028, -0.013)	0.0000
X - 25442	0.0003 (0.0001, 0.0005)	0.0208	0.011 (0.003, 0.019)	0.0107
(2,4 or 2,5)-dimethylphenol sulfate	0.0002 (0.0000, 0.0004)	0.0220	0.012 (0.004, 0.019)	0.0044
4-hydroxymandelate	-0.0002 (-0.0004, -0.0000)	0.0246	-0.013 (-0.020, -0.006)	0.0022
saccharin	0.0002 (0.0000, 0.0004)	0.0264	0.005 (-0.003, 0.012)	0.2566
hydantoin-5-propionate	-0.0002 (-0.0004, -0.0000)	0.0311	-0.004 (-0.011, 0.004)	0.3445
dopamine 4-sulfate	-0.0002 (-0.0004, -0.0000)	0.0318	-0.010 (-0.017, -0.002)	0.0208
X - 17825	0.0002 (0.0000, 0.0004)	0.0368	0.010 (0.003, 0.018)	0.0113
1-methylhistidine	0.0002 (0.0000, 0.0004)	0.0373	0.009 (0.003, 0.016)	0.0107
dopamine 3-O-sulfate	-0.0002 (-0.0004, -0.0000)	0.0430	-0.011 (-0.019, -0.004)	0.0080
2-ethylphenylsulfate	0.0002 (0.0000, 0.0004)	0.0580	0.010 (0.003, 0.017)	0.0102
4-hydroxycinnamate sulfate	-0.0002 (-0.0004, 0.0000)	0.0708	-0.013 (-0.021, -0.006)	0.0022
isoleucylhydroxyproline*	0.0002 (-0.0000, 0.0003)	0.0714	0.018 (0.011, 0.024)	0.0000
glycolate (hydroxyacetate)	0.0002 (-0.0000, 0.0004)	0.0754	0.016 (0.009, 0.022)	0.0001
4-acetamidobenzoate	-0.0002 (-0.0004, 0.0000)	0.0754	-0.008 (-0.016, -0.001)	0.0402
3,4-methylene heptanoylglycine	0.0002 (-0.0000, 0.0004)	0.0754	0.013 (0.005, 0.020)	0.0034
6-bromotryptophan	0.0002 (-0.0000, 0.0004)	0.0827	0.007 (-0.000, 0.014)	0.0715
X - 21258	-0.0002 (-0.0004, 0.0000)	0.0827	-0.007 (-0.015, 0.000)	0.0716
2S,3R-dihydroxybutyrate	-0.0002 (-0.0003, 0.0000)	0.0906	-0.007 (-0.014, -0.000)	0.0551
picolinoylglycine	-0.0002 (-0.0004, 0.0000)	0.1002	-0.010 (-0.017, -0.003)	0.0108
2-methylcitrate	-0.0001 (-0.0003, 0.0001)	0.2049	-0.008 (-0.015, -0.001)	0.0419

Models adjusted for age, sex, body mass index, energy intake, physical activity, alcohol and smoking status.
Abbreviations: β, regression estimate; CI, confidence intervals; FDR, false discovery rate according to Benjamini-Hochberg; TEI, total energy intake.
*Indicates a compound that has not been confirmed based on authentic chemical standard, but Metabolon are confident in its identity. The structural identities of 'X-' followed by a number (e.g., X - 11372) are unknown.

Supplemental Table 9. Regression estimates of the associations of absolute and energy-based UPF intake with urine metabolite patterns

Metabolite pattern	UPF (absolute intake, g/day)		% UPF TEI, β (95% CI)	
	**β (95%, CI)**	**FDR q-value**	**β (95%, CI)**	**FDR q-value**
**MP9**	**0.001 (0.000, 0.002)**	**0.0097**[1]	**0.042 (0.010, 0.075)**	**0.0146**[1]
MP10	0.001 (0.000, 0.002)	0.0102[1]	0.016 (-0.015, 0.046)	0.3071
**MP7**	-0.001 (-0.002, 0.000)	0.0956	**-0.075 (-0.108, -0.042)**	**0.0000**[1]
MP18	-0.001 (-0.001, 0.000)	0.1339	-0.042 (-0.070, -0.015)	0.0049[1]

[1]statistically significant results (FDR q-value <0.05).
Models adjusted for age, sex, body mass index, energy intake, physical activity, alcohol and smoking status.
Abbreviations: β, regression estimate; CI, confidence intervals; FDR, false discovery rate according to Benjamini-Hochberg; MP, metabolite pattern; TEI, total energy intake; UPF, ultra-processed foods

Supplemental Table 10. Regression estimates of the associations of absolute and energy-based UPF intake with plasma metabolites

Metabolite	UPF (absolute intake, g/day)		UPF (% energy contribution to TEI)	
	**β (95%, CI)**	**FDR q-value**	**β (95%, CI)**	**FDR q-value**
homostachydrine*	-0.0008 (-0.0012, -0.0003)	0.0022	-0.0316 (-0.0449, -0.0182)	0.0001
3-CMPFP	-0.0008 (-0.0012, -0.0003)	0.0022	-0.0151 (-0.0294, -0.0008)	0.0716
X - 11372	0.0007 (0.0003, 0.0011)	0.0022	0.0298 (0.0170, 0.0425)	0.0001
4-hydroxyglutamate	0.0006 (0.0003, 0.0010)	0.0036	0.0193 (0.0059, 0.0326)	0.0129
X - 24951	0.0007 (0.0003, 0.0011)	0.0036	0.0204 (0.0068, 0.0339)	0.0108
X - 13866	-0.0007 (-0.0011, -0.0003)	0.0036	-0.0136 (-0.0275, 0.0003)	0.0734
X - 23639	-0.0007 (-0.0011, -0.0003)	0.0036	-0.0224 (-0.0364, -0.0085)	0.0074
hydroquinone sulfate	-0.0007 (-0.0011, -0.0002)	0.0052	-0.0108 (-0.0253, 0.0037)	0.1645
betaine	-0.0004 (-0.0007, -0.0001)	0.0215	-0.0078 (-0.0188, 0.0032)	0.1722
X - 24337	0.0005 (0.0001, 0.0009)	0.0215	0.0168 (0.0033, 0.0303)	0.0342
phenol sulfate	-0.0005 (-0.0009, -0.0001)	0.0229	-0.0138 (-0.0274, -0.0003)	0.0716
methyl glucopyranoside (alpha + beta)	-0.0005 (-0.0009, -0.0001)	0.0229	-0.0224 (-0.0364, -0.0084)	0.0074
2-aminobutyrate	-0.0005 (-0.0009, -0.0001)	0.0296	-0.0117 (-0.0252, 0.0017)	0.1058
nonanoylcarnitine (C9)	-0.0004 (-0.0008, -0.0000)	0.0466	-0.0135 (-0.0270, -0.0000)	0.0716
X - 17357	-0.0004 (-0.0007, 0.0000)	0.0562	-0.0127 (-0.0251, -0.0003)	0.0716
X - 17340	-0.0003 (-0.0007, 0.0000)	0.0562	-0.0061 (-0.0179, 0.0058)	0.3134

Models adjusted for age, sex, body mass index, energy intake, physical activity, alcohol and smoking status, number of dietary assessments, and time difference between dietary assessment and blood draw.

*Indicates a compound that has not been confirmed based on authentic chemical standard, but Metabolon are confident in its identity. The structural identities of 'X-' followed by a number (e.g., X - 11372) are unknown.

Supplemental Table 11. Regression estimates of the associations of absolute and energy-based UPF intake with plasma metabolite patterns (*n* = 195)

**Metabolite pattern**	**UPF (g/d), β (95% CI)**		**% UPF TEI, β (95% CI)**	
	**β (95%, CI)**	**FDR q-value**	**β (95%, CI)**	**FDR q-value**
**MP8**	**0.002 (0.001, 0.004)**	**0.0207**	**0.113 (0.059, 0.167)**	**0.0002**
MP17	-0.001 (-0.003, -0.000)	0.0330	-0.033 (-0.073, 0.008)	0.1829
MP6	0.002 (0.000, 0.004)	0.0207	0.040 (-0.013, 0.094)	0.1828
MP1	-0.002 (-0.004, 0.000)	0.0726	-0.033 (-0.103, 0.037)	0.3585

Models adjusted for age, sex, body mass index, energy intake, physical activity, alcohol and smoking status, number of dietary assessments, and time difference between dietary assessment and blood draw.
Abbreviations: β, regression estimate; CI, confidence intervals; FDR, false discovery rate according to Benjamini-Hochberg; MP, metabolite pattern; TEI, total energy intake.

Supplemental Table 12. Regression estimates of the associations of UPF intake with urine metabolite patterns excluding potential underreporting (*n* = 260)

Metabolite pattern	% UPF (g/d), β (95% CI)	UPF (g/d), β (95% CI)	% UPF TEI, β (95% CI)
**MP7**	**-0.072 (-0.105, -0.039)[1]**	**-0.001 (-0.002, -0.000)[1]**	**-0.076 (-0.114, -0.039)[1]**
**MP9**	**0.053 (0.021, 0.085)[1]**	**0.002 (0.001, 0.003)[1]**	**0.051 (0.014, 0.088)[1]**
MP10	0.027 (-0.003, 0.058)	0.001 (0.000, 0.002)[1]	0.031 (-0.004, 0.066)
MP18	-0.037 (-0.064, -0.009)[1]	-0.001 (-0.002, 0.000)	-0.053 (-0.084, -0.022)[1]

[1]statistically significant results (FDR q-value <0.05).
Models adjusted for age, sex, body mass index, energy intake, physical activity, alcohol and smoking status.
Abbreviations: β, regression estimate; CI, confidence intervals; FDR, false discovery rate according to Benjamini-Hochberg; MP, metabolite pattern; TEI, total energy intake.

Supplemental Table 13. Regression estimates of the associations of UPF intake with plasma metabolite patterns excluding potential underreporting (*n* = 137)

Metabolite pattern	% UPF (g/d), β (95% CI)	UPF (g/d), β (95% CI)	% UPF TEI, β (95% CI)
MP1	-0.065 (-0.121, -0.009)[1]	-0.002 (-0.004, 0.000)	-0.035 (-0.114, 0.045)
MP6	0.049 (0.004, 0.094)[1]	0.002 (0.000, 0.004)[1]	0.069 (0.006, 0.133)
**MP8**	**0.078 (0.031, 0.126)[1]**	**0.002 (0.000, 0.004)[1]**	**0.138 (0.073, 0.203)[1]**
MP17	-0.031 (-0.066, 0.004)	-0.001 (-0.002, 0.000)	-0.019 (-0.069, 0.030)

[1]statistically significant results (FDR q-value <0.05).
Models adjusted for age, sex, body mass index, energy intake, physical activity, alcohol and smoking status, number of dietary assessments, and time difference between dietary assessment and blood draw.

Abbreviations: β, regression estimate; CI, confidence intervals; FDR, false discovery rate according to Benjamini-Hochberg; MP, metabolite pattern.

References

Monteiro CA, Cannon G, Levy RB, Moubarac JC, Louzada ML, Rauber F, et al. Ultra-processed foods: what they are and how to identify them. Public Health Nutr. 22 (2019), 936–941.
